# A biologically structured hierarchical vision transformer-CNN framework for robust tomato leaf disease classification

**DOI:** 10.3389/frai.2026.1880070

**Published:** 2026-08-05

**Authors:** Harshinisree Gunasekaran, Sujatha Rajkumar, B. Lincy Kirubhadharsini

**Affiliations:** 1 School of Biosciences and Technology (SBST), Vellore Institute of Technology (VIT), Vellore, India; 2 VIT School of Electronics Engineering (SENSE), Vellore Institute of Technology (VIT), Vellore, India; 3 VIT School of Agricultural Innovations and Advanced Learning (VAIAL), Vellore Institute of Technology (VIT), Vellore, India

**Keywords:** EfficientNet, hierarchical deep learning, robustness analysis, tomato leaf disease, vision transformer

## Abstract

Precise and reliable diagnosis of leaf diseases in tomato is essential for enhancing crop cultivation and minimizing agricultural losses. While deep learning models have performed well on benchmark datasets, the majority of present techniques rely on flat multi-class classification, which predicts all disease categories simultaneously. Such formulations promotes inter-class confusion, particularly when biologically different diseases with similar visual symptoms are learned within a same model. To overcome this constraint, we propose a biologically structured hierarchical deep learning framework in this study. Instead of directly classifying 10 disease classes, the proposed method first classifies leaf images into meaningful biological groups such as bacterial, fungal, pest-associated and healthy using a vision transformer (ViT) model. Then, specialized convolutional neural network (CNN) experts perform fine-grained classification within each category. The proposed hierarchical model shows an overall accuracy of 97.8%, when validated on PlantVillage tomato dataset. A flat ViT model trained with class-weighted loss obtained 96.3% accuracy, whereas a flat CNN model reached 99.3% under clean conditions but decreased sharply to 41% under Gaussian perturbation (*σ* = 0.05). On the other hand, the hierarchical model performed steadily under noise with 97.4% accuracy at the same perturbation level. These results indicate that adding biological structure to model design reduces confusion, helps prevent imbalance effects and increases robustness, providing a more trustworthy and interpretable solution for real-world agricultural disease diagnosis.

## 1 Introduction

Tomato (*Solanum lycopersicum*) is one of the most economically valued vegetable crop in the world, contributing significantly to the economy of agriculture and global food safety. But, tomato crops are extremely prone to foliar diseases caused by fungal pathogens, bacterial infections and pest-associated viral organisms. These diseases have the potential to significantly reduce yield and quality, if they are not identified in time. In traditional disease diagnosis, disease detection is dependent on visual analysis by domain specialists which is tedious, inaccurate and unfeasible in large-scale farming systems.

In recent years, deep learning has improved plant disease detection by automating it significantly. Early research proved that deep convolutional neural networks could accurately detect plant leaf diseases from RGB images, laying the groundwork for image-based plant pathology (Sladojevic et al., 2016). Subsequent research further validated CNN-based approaches using pretrained architectures and transfer learning strategies on benchmark datasets such as PlantVillage (Chaudhry, 2021), reporting high classification accuracy (Too et al., 2019; Ferentinos, 2018; Mohanty et al., 2016). Moreover, transfer learning with pretrained architectures like ResNet and EfficientNet has increased classification accuracy and computational efficiency (Chen et al., 2020; Tan and Le, 2019). Kamilaris and Prenafeta-Boldú (2018) found that deep learning has a revolutionary impact on agriculture. Despite reporting high clean-test accuracy, most present methods treat plant disease diagnosis as a simple multi-class classification problem.

Vision transformers (ViTs) have been recently emerged as an alternative to convolution-based architectures by using self-attention mechanisms to model global contextual relationships (Dosovitskiy et al., 2021; Touvron et al., 2021). Moreover, these transformer-based models are used in plant disease classification tasks showing promising results, which indicates the capability of attention-based architectures to capture global contextual features in leaf images (Mookkandi et al., 2025; Saleem et al., 2019). Their ability to detect long-range dependencies suggests that they may be robust under visual variability.

Although these advances have several benefits but they also have certain limitations. First, flat classification systems ignore biological taxonomy, despite the fact that fungal, bacterial, and pest-associated diseases exhibit differences in pathological manifestation. Second, plant disease datasets commonly show class imbalance which is frequently addressed by cost-sensitive loss functions such as focal loss or class-balanced loss (Lin et al., 2017; Cui et al., 2019; Johnson and Khoshgoftaar, 2019). Third, despite recent studies showing persistent difficulties in creating reliable and broadly applicable deep-learning based plant disease detection systems, robustness under distributional perturbations such as noise, domain shift and illumination variation has received less attention in agricultural contexts (Hendrycks and Dietterich, 2019; Geirhos et al., 2019; Shoaib et al., 2023).

To address these constraints, our review suggests a biologically structured hierarchical deep learning framework for identifying tomato leaf diseases. The suggested method divides the classification problem into two stages: one is transformer-based biological routing and the other one is CNN-based expert specialization. By combining model architecture with disease etiology, the methodology reduces inter-class confusion and simplifies decision boundaries. Additionally, model stability is evaluated through a systematic robustness evaluation under controlled Gaussian perturbations. The proposed approach demonstrates enhanced robustness while retaining competitive accuracy when compared to flat CNN and flat vision transformer model.

## 2 Related works

### 2.1 CNN-based classification of plant diseases

Mostly plant disease detection rely on convolutional neural network based systems due to their capacity to extract discriminative features from leaf images automatically. Mohanty et al. (2016) and Too et al. (2019) conducted systematic analysis of pretrained CNN architectures and found that deeper residual networks outperformed shallower counterparts in plant disease recognition tasks. Barbedo (2018, 2019) highlighted that how CNN performance gets affected by the effect of dataset variability and environmental conditions. Moreover, studies by Arsenovic et al. (2019) and Ghosal et al. (2018) have highlighted the significance of field deployment and dataset variability. Chen et al. (2020) showed that efficientNet-based transfer learning improves computational efficiency while maintaining high classification performance. Recent work on CNN models that includes attention mechanisms, improved feature aggregation and lightweight architectures have strong performance on both controlled and real-world plant disease datasets, which increases robustness and deployment potential (Karthikeyan et al., 2025; Arshad et al., 2026; Goyal et al., 2026). Additionally, current studies have emphasized the expanded use of explainable AI (XAI) and edge-focused architectures and lightweight CNNs for practical tomato disease diagnosis, which highlights their importance in real-world agricultural applications (Gunasekaran et al., 2026).

Though CNNs perform well in extracting hierarchical local features, they rely primarily on localized receptive fields and are typically implemented as flat multi-class classifiers, which increases decision boundary complexity across etiologically diverse diseases.

### 2.2 Transformer-based models in agricultural vision

Vision transformers revolutionized image recognition by replacing convolution operations with patch-based self-attention (Dosovitskiy et al., 2021). Data-efficient training strategies such as DeiT, enhanced transformer performance on moderate-sized datasets (Touvron et al., 2021). Mookkandi et al. (2025) proposed a lightweight vision transformer architecture for crop disease classification, demonstrating improved robustness and computational efficiency over conventional CNN-based approaches, while hybrid CNN-transformer models have been investigated to combine local and global feature modeling (Saleem et al., 2019). PlantXViT (Thakur et al., 2022), ConvTransNet-S (Jia et al., 2025) and hybrid CNN-Vision Transformer frameworks proposed by Aboelenin et al. (2025) are few examples that have demonstrated strong performance in plant disease recognition by utilizing the complementary strengths of convolutional and attention-based representations. However, most transformer-based agricultural models have flat classification structures and do not include hierarchical biological relationships.

### 2.3 Class imbalance and cost-sensitive learning

In plant disease datasets, class imbalance is a recurring problem. Focal loss (Lin et al., 2017) and class-balanced loss (Cui et al., 2019) adjust training objectives to prioritize minority classes. Johnson and Khoshgoftaar (2019) conducted a detailed assessment of deep learning under class imbalance conditions. While such strategies increase minority-class recall, they do not effectively eliminate the cross-category interference that comes with flat classification structures.

### 2.4 Hierarchical and expert-based classification

Hierarchical classification breaks down complex multi-class classification tasks into structured stages. Hierarchical decomposition which separates coarse and fine-grained identification tasks can simplify decision boundaries and reduce inter-category confusion in complex classification problems (Silla and Freitas, 2011; Wehrmann et al., 2018). In order to increase scalability and specialization, mixture of expert models dynamically route inputs to specialized sub network (Shazeer et al., 2017). By allowing individual subnetworks to focus on specific subset of classes, expert specialization encourages discriminative feature learning while reducing competition among unrelated categories (Fedus et al., 2022). Although hierarchical frameworks have proven successful in other computer vision applications, biologically structured decomposition is still underexplored in plant disease classification. A recent work by Iwano et al. (2024) to enhance plant disease diagnosis has proposed a hierarchical object detection and recognition framework (HODRF) by combining object detection and classification. Their work suggested the need for biologically organized classification strategies by highlighting the drawbacks of traditional flat single classification techniques and showing how hierarchical breakdown might increase diagnostic accuracy while lowering false positives.

### 2.5 Robustness and distribution shift

For real-world agricultural deployment of deep learning models, robustness under distributional perturbation is essential. In order to assess the robustness of neural network, Hendrycks and Dietterich (2019) developed corruption benchmarks. Geirhos et al. (2019) found that CNNs frequently rely on textural bias, which makes them susceptible to perturbations. Moreover, it has been demonstrated that domain variability causes performance degradation under real-field conditions (Singh et al., 2020; Arsenovic et al., 2019). But still, systematic perturbation-based robustness analysis has limited application in tomato leaf disease classification.

### 2.6 Research gap

The studied literature reveals three main limitations:

1 A significant reliance on flat multi-class classification without biological structuring.
2 There is a limited integration of hierarchical taxonomy into model architecture.
3 Inadequate robustness analysis under controlled perturbations.

Although CNN and transformer architectures have shown good discriminative performance, structural decomposition based on disease etiology has not been thoroughly examined for tomato leaf disease classification.

To overcome these constraints, the present study proposes a biologically structured hierarchical framework that combines transformer-based routing with CNN-based expert specialization, which reduces inter-class confusion and improves robustness under distributional perturbations.

## 3 Methodology

### 3.1 Problem formulation

Tomato leaf disease classification is conventionally defines as a supervised multi-class image classification problem. Given an input RGB image $x\;\in \;{\mathbb{R}}^{H\times W\times 3}$ , the goal is to learn a mapping function as defined in Equation (1):


$$f_{1}:{\mathbb{R}}^{H\times W\times 3}\to \{1,2,\ldots ,K\}$$ (1)

where, *K* = 10 represents the total disease classes.

The model outputs a probability distribution for all classes as given in Equations (2) and (3):


$$p=[p_{1},p_{2},\ldots ,p_{K}]$$ (2)

where:


$$p_{c}=\frac{e^{\mathrm{zc}}}{\sum_{j=1}^{K}e^{\mathrm{zj}}}$$ (3)

here, *z_c_* represents the logit associated with class *c*, and *p_c_* represents the predicted probability for class *c*. The soft-max function ensures that $\sum_{c=1}^{K}p_{c}=1.$

The predicted class is shown in Equation (4):


$$\hat{y}=\arg\max_{c}p_{c}$$ (4)

Although this flat formulation is straightforward, it needs a single model to simultaneously distinguish between all disease classes, even though they belong to biologically different disease groups (fungal, bacterial, pest-associated and healthy). This complicates decision boundaries and causes inter-class confusion, especially when visual symptoms overlap between different disease groups.

To study and address this structural limitation, both flat baseline models and a biologically structured hierarchical framework were developed.

### 3.2 Class-weighted learning for flat baseline models

Class imbalance is the major challenge in plant disease datasets, where some disease classes have significantly more training samples than others. In such cases, a standard cross-entropy loss function may bias the model toward majority classes.

The standard cross-entropy loss is given in Equation (5):


$$L_{\mathrm{CE}}=-\sum_{c=1}^{K}y_{c}\log(p_{c})$$ (5)

where $y_{c}\epsilon \{0,1\}$ indicates whether the sample belongs to class *c* and *p_c_* is the predicted probability for class *c*.

To mitigate imbalance, a class-weighted cross-entropy loss was employed in flat baseline experiments. The weight assigned to class *c* was computed using Equation (6):


$$w_{c}=\frac{N}{K.n_{c}}$$ (6)

where *N* denotes total number of training samples, *K* represents total number of classes and *n_c_* denotes number of samples belonging to class *c*.

The weighted loss is expressed in Equation (7):


$${ℒ}_{\mathrm{WCE}}=-\sum_{c=1}^{K}w_{c}y_{c}\;\log(p_{c})$$ (7)

This formulation increases the penalty for misclassification of minority classes while decreasing the dominance of majority categories during optimization.

Although class-weighted learning overcomes the imbalance, it does not change the structural formulation of the classification problem. The model continues to learn all 10 disease classes simultaneously and cross-category confusion may remain.

### 3.3 Biologically structured hierarchical framework

A biologically guided hierarchical architecture was proposed to reduce inter-class confusion and simplify decision complexity. Instead of predicting one of ten disease classes, the classification problem is divided into two stages based on biological taxonomy.

#### 3.3.1 Stage 1: biological category routing

In first stage, vision transformer (ViT routing model - DeiT-Tiny) is learned to classify input images into four biologically meaningful categories, that is bacterial, fungal, pest-associated and healthy.This routing function, the probability vector and the predicted category is given in Equations (8), (9) and (10).


$$f_{1}:{\mathbb{R}}^{H\times W\times 3}\to \{1,2,3,4\}$$ (8)

The routing stage outputs:


$$P^{\left(1\right)}=[P_{1}^{\left(1\right)},P_{2}^{\left(1\right)},P_{3}^{\left(1\right)}P_{4}^{\left(1\right)}]$$ (9)

The predicted category is:


$$\hat{c}=\arg\max_{i}p_{i}^{\left(1\right)}$$ (10)

By initially defining the broader disease etiology, this stage narrows down the search space for future fine-grained classification.

#### 3.3.2 Stage 2: expert subnetworks

Based on the predicted category $\hat{c}$ , a category-specific expert CNN performs fine-grained classification.

For example:

- If $\hat{c}$ = Fungal, a 5-class EfficientNet-B0 model is activated.
- If $\hat{c}$ = Pest-associated, a 3-class EfficientNet-B0 model is activated.
- Bacterial and Healthy categories correspond to single-class mappings.

Formally, the final prediction is expressed in Equation (11):


$$f(x)=f_{2}^{\left(\hat{c}\right)}(f_{1}(x))$$ (11)

where, $f_{1}(x)$ is the routing function and $f_{2}^{\left(\hat{c}\right)}$ is the expert model corresponding to category $\hat{c}$ .

**Figure 1:**
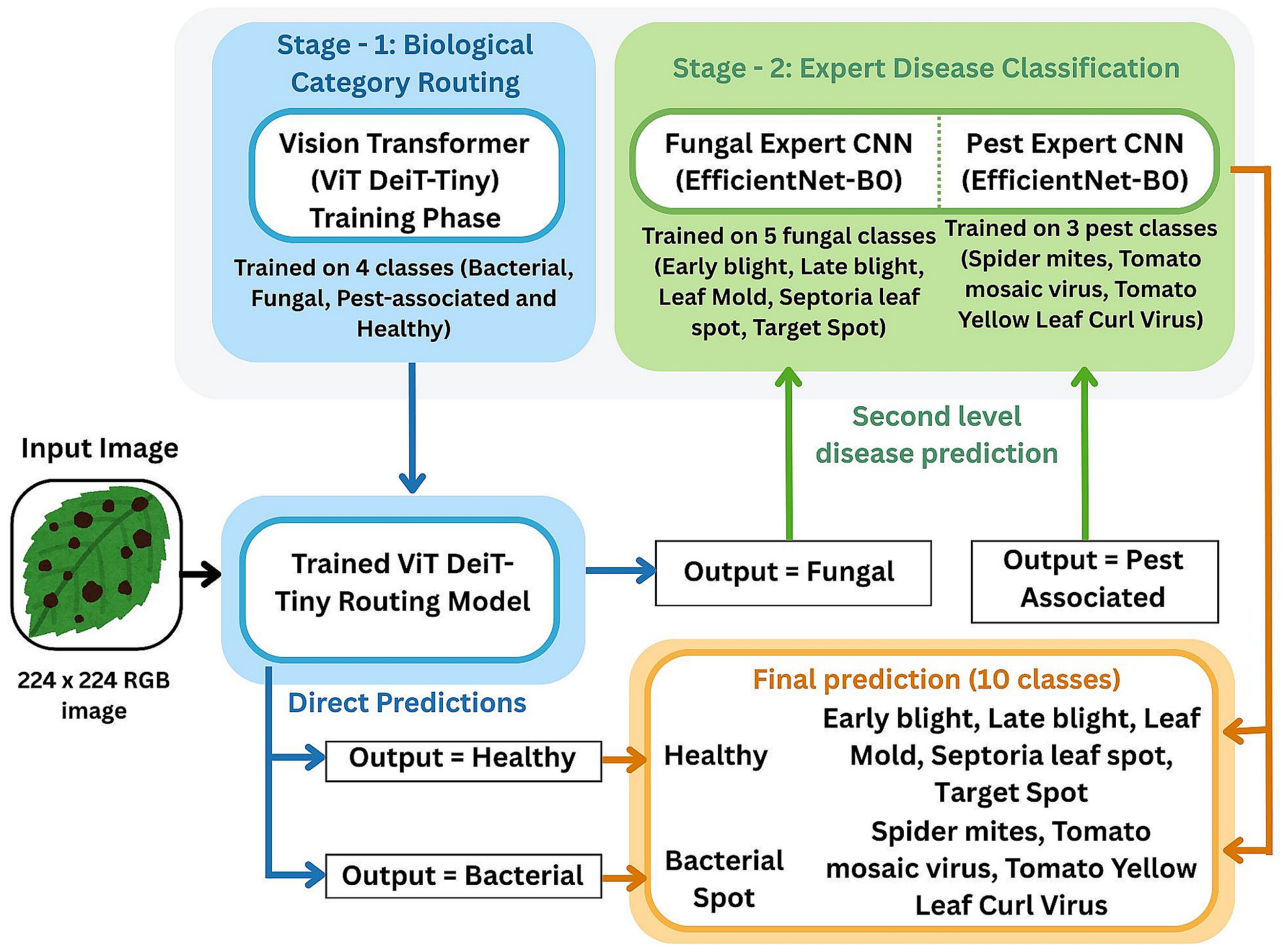
Overview of proposed biologically structured hierarchical framework for tomato leaf disease classification. A vision transformer performs biological category routing at stage-1, followed by fine-grained disease classification by category-specific CNN experts performance.

The routing stage uses a vision transformer to differentiate biological categories based on global contextual information. In contrast, CNN-based expert networks are used for fine-grained disease classification because disease-specific lesion patterns and texture characteristics are mostly local visual features that can be efficiently captured by convolutional architectures. This decomposition simplifies the decision boundaries by distinguishing intra-category discrimination from inter-category separation. Figure 1 depicts the hierarchical routing and expert structure.

### 3.4 Robustness modeling

In real-time agricultural situations, illumination changes, sensor noise and environmental disturbances can all have an impact on image acquisition. To assess model stability under such perturbations, robustness experiments were conducted with additive gaussian noise.

For a normalized image tensor *x*, noisy input was generated according to Equations (12) and (13):


$$x_{\text{noisy}}=x+\epsilon$$ (12)

where:


$$\in ∼N(0,\sigma^{2})$$ (13)

To assess the robustness of the model, two levels of Gaussian noise were taken into consideration: a mild perturbation with *σ* = 0.02 and a strong perturbation with *σ* = 0.05.

In order to maintain feature distributions consistent with training conditions, noise was directly applied to normalized tensors without clamping.

### 3.5 Performance evaluation metrics

The classification performance of proposed hierarchical framework and flat baseline models was assessed with standard metrics from confusion matrix which includes accuracy, precision, recall and *F*1-score. To provide a deeper analysis, additional evaluation metrics such as balanced accuracy, Matthews correlation coefficient (MCC) and Cohen’s kappa are also calculated. These metrics helps in understanding the model performance, especially in multi-class classification problems where class imbalance may influence the interpretation of results.

In multi-class classification, confusion matrix describes the prediction results by comparing the ground truth labels and the predicted class labels. This matrix is used to get true positive (TP), false positive (FP), true negative (TN) and false negative (FN) for each class using one-versus-rest approach, allowing for the calculation of standard performance metrics. All the standard metrics were defined in Equations (14)–(20).

Accuracy is the measure of percentage of correctly categorized samples across all predictions and denoted by:


$$\text{Accuracy}=\frac{\mathrm{TP}+\mathrm{TN}}{\mathrm{TP}+\mathrm{TN}+\mathrm{FP}+\mathrm{FN}}$$ (14)

Precision quantifies proportion of correctly predicted positive samples across all positive predictions:


$$\text{Precision}=\frac{\mathrm{TP}}{\mathrm{TP}+\mathrm{FP}}$$ (15)

Recall, also known as sensitivity quantifies the percentage of actual positive samples that are correctly classified by the model:


$$\text{Recall}=\frac{\mathrm{TP}}{\mathrm{TP}+\mathrm{FN}}$$ (16)

The *F*1-score denotes harmonic mean of precision and recall:


$$F1=2\times \frac{\text{Precision}\times \text{Recall}}{\text{Precision}+\text{Recall}}$$ (17)

Balanced accuracy measures the average recall obtained across all classes and is defined as:


$$\text{Balanced Accuracy}=\frac{\left(\text{Sensitivity}+\text{Specificity}\right)}{2}$$ (18)

where, sensitivity is referred as TP/(TP + FN) and specificity is represented by TN/(TN + FP)

Matthews correlation coefficient (MCC) gives a balanced evaluation by considering true positive, true negative, false positive and false negative simultaneously:


$$\mathrm{MCC}=\frac{\mathrm{TP}\times \mathrm{TN}-\mathrm{FP}\times \mathrm{FN}}{\sqrt{\left(\mathrm{TP}+\mathrm{FP})(\mathrm{TP}+\mathrm{FN})(\mathrm{TN}+\mathrm{FP})(\mathrm{TN}+\mathrm{FN}\right)}}$$ (19)

Cohen’s kappa quantifies the degree of agreement between predicted and actual labels while taking chance into consideration:


$$k=\frac{P_{o}-P_{e}}{1-P_{e}}$$ (20)

where $P_{o}$ refers to observed agreement and $P_{e}$ indicates the expected agreement by chance.

Additionally, 95% confidence intervals were calculated for classification accuracy to evaluate the statistical reliability and stability of the provided performance estimates. For the multi-class evaluation, macro-averaged and weighted-averaged measures were calculated across all disease categories. Macro-averaging treats each class equally by taking the average of class-wise metrics whereas weighted-averaging takes the amount of samples in each class. Both averaging techniques were employed to give a thorough and impartial evaluation of model performance, because the PlantVillage tomato dataset exhibits class imbalance across disease categories. In addition to quantitative metrics, confusion matrix analysis was used to visually represent class-wise prediction behavior and highlight probable misclassification patterns across disease categories.

## 4 Experimental setup

### 4.1 Dataset description and partitioning

The experiments were carried out on the publicly available PlantVillage tomato leaf disease dataset (Chaudhry, 2021). The dataset contains 14,529 RGB images divided into 10 categories, which includes fungal diseases, bacterial infections, pest-associated viral diseases, and healthy leaves. Table 1 lists the detailed distribution of the dataset. As observed, the dataset has a considerable imbalance between categories particularly in the pest-associated viral disease class.

**Table 1:** Class distribution of PlantVillage tomato leaf dataset.

S. no	Disease class	Biological category	Number of images
1	Tomato___Early_blight	Fungal	800
2	Tomato___Late_blight	Fungal	1,527
3	Tomato___Leaf_Mold	Fungal	761
4	Tomato___Septoria_leaf_spot	Fungal	1,417
5	Tomato___Target_Spot	Fungal	1,123
6	Tomato___Bacterial_spot	Bacterial	1702
7	Tomato___Spider_mites Twospotted_spider_mite	Pest-associated	1,341
8	Tomato___Tomato_mosaic_virus	Pest-associated	299
9	Tomato___Tomato_Yellow_Leaf_Curl_Virus	Pest-associated	4,286
10	Tomato___healthy	Healthy	1,273
Total			14,529

The dataset was divided into training (70%), validation (15%) and testing (15%) using deterministic random splitting, with a fixed seed of 42 to assure reproducibility. To ensure fair comparison and consistency, the same partition was used for both flat and hierarchical experiments.

### 4.2 Image preprocessing

To meet the input requirements of EfficientNet-B0 and DeiT-Tiny architectures, all images were resized to 224 × 224 pixels. Images were transformed into tensors and normalized with ImageNet mean and standard deviation values as shown in Equation (21):


$$x_{\mathrm{norm}}=\frac{x-\mu }{\sigma }$$ (21)

where, *μ* = [0.485, 0.456, 0.406] and *σ* = [0.229, 0.224, 0.225].

No additional geometric data augmentation was applied, allowing the robustness analysis to remain controlled and directly attributable to Gaussian perturbations defined in Section 3.4.

### 4.3 Computational environment

All experiments are done in google colab utilizing NVIDIA Tesla T4 GPU acceleration (16 GB VRAM). The implementation was built in *Python 3* with the *PyTorch* deep learning framework. Pretrained CNN architectures were accessed through *torchvision* and transformer-based models were implemented using the *timm* library.

To maintain experimental reproducibility, the random seed was set to 42 for Python, NumPy and PyTorch. Moreover, In addition, cuDNN was set to deterministic mode with non-deterministic backend optimizations disabled, to ensure consistent model training evaluation. This configuration assures steady training behavior across runs and consistent data splits.

### 4.4 Model initialization and training configuration

To leverage transfer learning and speed up convergence, all models were set up with ImageNet-pretrained weights. AdamW optimizer was used at an initial learning rate of 3 × 10^−4^ and a batch size of 32 for optimization. Each model was trained for 10 epochs with validation performance assessed at the end of each epoch.

Flat baseline models used weighted cross-entropy loss as explained in Section 3.2. In contrast, standard cross-entropy loss was used for the proposed hierarchical framework (Stage-1 routing and Stage-2 experts), including both the routing model and the category-specific expert networks. Compared to the flat 10-class classification task, the hierarchical architecture breaks down the problem into biologically meaningful subsets, which in turn reduces the class imbalance within the fungal expert network and simplifying the learning task for the expert models. Although some imbalance persisted within the pest subset, the expert networks achieved high classification accuracies, showing that the hierarchical decomposition itself effectively solved much of the imbalance-related challenge. Table 2 summarizes the primary training hyper parameters used.

**Table 2:** Training configuration parameters.

S. no	Parameter	Value
1	Optimizer	AdamW
2	Learning rate	3 × 10^−4^
3	Batch size	32
4	Number of epochs	10
5	Pretraining	ImageNet
6	Loss (flat baselines)	Weighted cross-entropy
7	Loss (hierarchical stages)	Cross-entropy
8	Input resolution	224 × 224

### 4.5 Hierarchical configuration details

In the hierarchical architecture, the stage-1 ViT routing model (DeiT-Tiny) was trained to classify images into four biological categories. The stage-2 expert networks were then trained independently on category-specific subsets:

- An EfficientNet-B0 model with 5 classes for fungal diseases.
- An EfficientNet-B0 model with 3 classes for pest-associated diseases.

Since bacterial and healthy categories does not have sub-classes, they are directly mapped after stage-1 without the need for stage-2 sub-networks. To maintain uniformity, all hierarchical components were trained using same optimization parameters listed in Table 2.

## 5 Results and discussion

Experimental evaluation was performed to analyze (i) convergence behavior, (ii) classification performance under clean conditions and (iii) robustness against controlled Gaussian perturbations. To ensure direct comparison, all the models were tested on the same test set containing 2,180 images. To assist the readability of the paper, the confusion matrices for all evaluated models are given in Supplementary Figures S1–S6.

### 5.1 Convergence behavior

**Figure 2:**
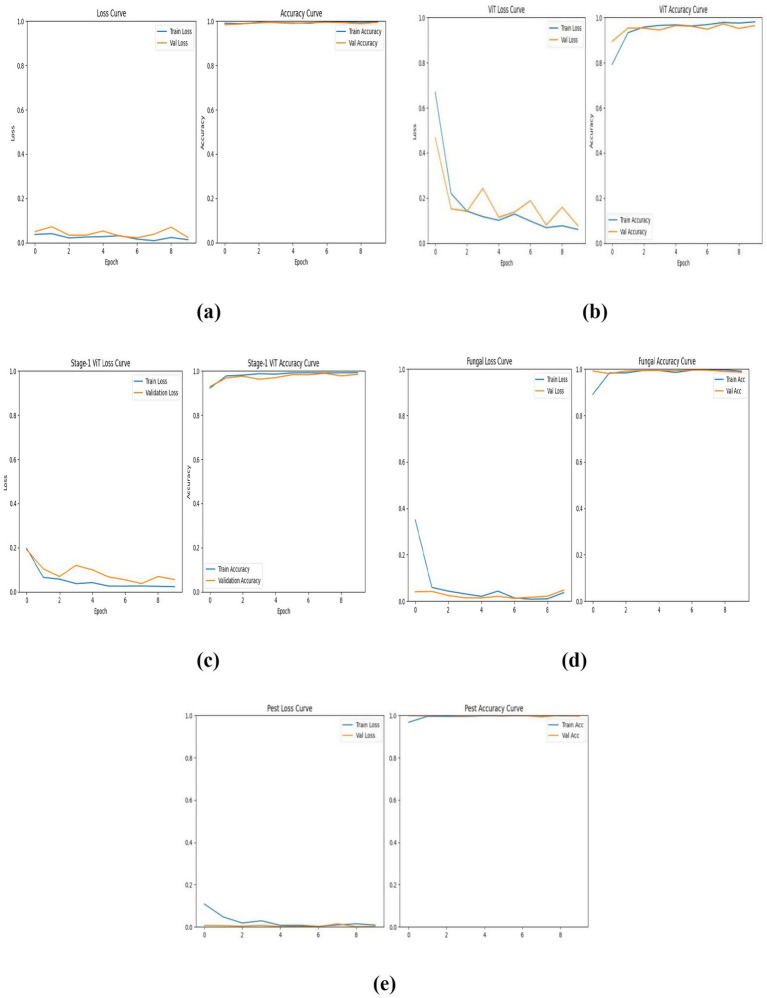
Training and validation dynamics (loss and accuracy curves), for **(a)** flat CNN model, **(b)** flat ViT model, **(c)** stage-1 ViT routing model, **(d)** fungal expert CNN model, and **(e)** pest expert CNN model.

The flat CNN model shows quick convergence with training loss falling rapidly within the first three epochs and then stabilizing below 0.05. Validation loss shows less overfitting since it closely tracks the training curve without diverging. Training and validation accuracies approach 99% early in training and remain consistent throughout consecutive epochs. Figure 2a illustrates the training and validation dynamics of the flat CNN model.

On the other hand, the flat ViT model shows a slightly more gradual convergence, as seen in Figure 2b. While validation accuracy closely follows training performance, training accuracy slowly increases from around 80 to 96% within four epochs. Although minor fluctuations in validation loss are found in subsequent epochs, there is no evidence of systematic overfitting. Overall, over the ten-epoch training period, both architectures show steady optimization behavior.

The convergence behavior of the hierarchical model is almost similar to flat CNN and flat ViT model. As per Figure 2c, the stage-1 ViT routing model runs consistently without oscillation and achieves 98% validation accuracy within a few epochs. The fungal expert CNN (Figure 2d) shows reduced intra-category decision complexity by rapidly approaching near-perfect accuracy. Whereas, the pest expert CNN (Figure 2e) achieves faster convergence, with 100% validation accuracy in the early epochs and maintaining zero validation loss. The training and validation curves are well aligned across all hierarchical components, showing effective generalization and absence of overfitting.

After establishing steady convergence behavior across all models, the classification performance and robustness under clean and perturbed conditions are evaluated in the subsequent sections.

### 5.2 Flat CNN model performance

#### 5.2.1 Clean test evaluation

Under clean test conditions (without adding noise), the flat CNN model achieved accuracy of 99.3%. The weighted *F*1-score was 0.993 while macro-averaged precision, recall, and *F*1-score were 0.991, 0.992, and 0.991, respectively. These findings are summarized in Table 3, which show highly discriminative class boundaries under controlled conditions. The majority of categories had per class *F*1-score greater than 0.98, with tomato leaf mold class exhibiting perfect classification. These results confirm that convolutional architectures can achieve near-perfect accuracy on curated benchmark datasets.

**Table 3:** Performance of flat CNN model under clean and noisy conditions.

Metric	Clean	*σ* = 0.02	*σ* = 0.05
Accuracy	0.993	0.923	0.410
Macro precision	0.991	0.931	0.620
Macro recall	0.992	0.927	0.450
Macro *F*1-score	0.991	0.917	0.350
Weighted precision	0.993	0.951	0.760
Weighted recall	0.993	0.923	0.410
Weighted *F*1-score	0.993	0.926	0.410
Test samples	2,180	2,180	2,180

#### 5.2.2 Mild Gaussian perturbation (*σ* = 0.02)

The flat CNN model’s overall accuracy dropped to 92.3%, when tested under mild Gaussian noise (*σ* = 0.02), with macro *F*1-score reduced to 0.917 and weighted *F*1-score to 0.926 (Table 3).

The recall of bacterial_spot class has dropped significantly and for certain fungal subclasses exhibiting increased misclassification among visually similar lesions. Despite the model’s very good accuracy, the decrease in macro recall shows that minority or subtle classes are more sensitive to perturbation. It shows the emerging inter-class confusion which reflects the degradation in the classification performance.

#### 5.2.3 Strong Gaussian perturbation (*σ* = 0.05)

When the flat CNN model was tested under stronger perturbation (*σ* = 0.05), it degraded substantially. Overall accuracy decreased drastically to 41.0%, with macro *F*1-score of 0.35 and weighted *F*1-score to 0.41, as shown in Table 3. It reveals the widespread misclassification across all disease classes.

Importantly, recall for bacterial_spot reached zero, while early_blight, septoria_leaf_spot, and target_spot showed severe cross-class confusion. This significant collapse implies that the CNN is strongly reliant on localized texture-based features which become unstable under noise perturbation. Although clean accuracy is exceptionally good, the model has low robustness under distributional shift. This comparison should be taken in consideration of the fact that flat CNN baseline was trained without image augmentation, which is the most common method to improve robustness against distribution shift. The corresponding confusion matrices is provided in Supplementary Figure S1.

### 5.3 Flat ViT model performance

#### 5.3.1 Clean test evaluation

The flat ViT model resulted in an overall clean test accuracy of 96.3%, with macro *F*1-score of 0.958 and weighted *F*1-score of 0.964 (Table 4). Although the model performance is slightly lower than the flat CNN model’s clean performance, the class-wise metrics show balanced behavior across categories. This shows some uncertainty across few fungal subclasses while maintaining consistently high recall for the majority of classes. The results reveal that transformer-based global attention modeling achieves competitive discriminative performance even under flat classification.

**Table 4:** Performance of flat vision transformer model under clean and noisy conditions.

Metric	Clean	*σ* = 0.02	*σ* = 0.05
Accuracy	0.963	0.962	0.948
Macro precision	0.957	0.956	0.942
Macro recall	0.961	0.960	0.945
Macro *F*1-score	0.958	0.957	0.940
Weighted precision	0.966	0.965	0.955
Weighted recall	0.963	0.962	0.948
Weighted *F*1-score	0.964	0.962	0.949
Test samples	2,180	2,180	2,180

#### 5.3.2 Mild Gaussian perturbation (*σ* = 0.02)

The flat ViT model maintained almost equal performance under mild perturbation (*σ* = 0.02), reaching 96.2% accuracy with a macro *F*1-score of 0.957 (Table 4). These results indicate that a very little structural degradation in class discrimination and is almost identical to the clean condition. This stability is significantly different from the CNN baseline implying that self-attention mechanisms may be less sensitive to localized noise.

#### 5.3.3 Strong Gaussian perturbation (*σ* = 0.05)

The flat ViT model had an overall accuracy of 94.8% with macro *F*1-score of 0.940 and weighted *F*1-score of 0.949 (Table 4), when tested under higher Gaussian noise (*σ* = 0.05). The decrease in accuracy is less when compared to the clean condition and does not indicate catastrophic failure. There is a low performance variation between *σ* = 0.02 and *σ* = 0.05. The persistent performance under perturbation demonstrates the robustness of global self-attention mechanisms, which capture long-range contextual relationships rather than depending just on fine-grained texture cues.

The corresponding confusion matrices is provided in Supplementary Figure S2.

### 5.4 Hierarchical framework evaluation

#### 5.4.1 Stage-1 ViT routing model performance

The stage-1 ViT routing model, which was trained to classify images into four biological categories (Bacterial, Fungal, Pest-associated and Healthy), performed consistently across noise levels. Under clean test conditions, the routing model achieved 97.98% accuracy, with a macro *F*1-score of 0.980 and weighted *F*1-score of 0.980. This results in substantial diagonal dominance, indicating low inter-category confusion. The largest fungal class achieved 0.978 *F*1-score, whereas the healthy achieved 0.997 *F*1-score, indication good biological separability.

The routing accuracy slightly increased to 98.03% under mild Gaussian perturbation (*σ* = 0.02), while the macro *F*1-score remained at 0.981. Similarly, routing accuracy stayed constant at 97.9% under higher perturbation (*σ* = 0.05), with slight variation in macro and weighted averages. Most importantly, no catastrophic routing failure is observed. This stability is crucial because hierarchical classification depends on proper routing. These findings shows that flat ten-class discrimination is not as robust as coarse biological discrimination.

The corresponding confusion matrices is provided in Supplementary Figure S3.

#### 5.4.2 Fungal expert CNN model performance

The fungal expert CNN model, responsible for distinguishing the five fungal diseases, achieved a clean test accuracy of 99.42% and macro F1-score of 0.994. This indicates that near-perfect intra-category discrimination, with only minimal confusion between the fungal classes, early_blight and late_blight. This implies that when the problem space is reduced to biologically consistent subclasses, decision boundaries become much simpler.

Under mild perturbation (*σ* = 0.02), accuracy remained incredibly high at 99.07%, with macro *F*1-score of 0.990. Under higher perturbation (*σ* = 0.05), performance decreased slightly to 98.37%, with macro *F*1-score of 0.981. Notably, Importantly, no structural collapse is detected and confusion remains localized among visually similar fungal subclasses rather than unrelated categories.

Compared to the flat CNN model, which collapsed after intense perturbation, the fungal expert is remarkably robustness. This shows that isolating intra-category classification minimizes cross-class interference while increasing feature stability.

The corresponding confusion matrices is provided in Supplementary Figure S4.

#### 5.4.3 Pest expert CNN model performance

The pest expert CNN model achieved a perfect classification under clean conditions, with 100% accuracy and an F1-score of 1.00 across all three pest-related classes. The corresponding confusion matrix is provided in Supplementary Figure S5.

Interestingly, under both *σ* = 0.02 and *σ* = 0.05 perturbations, the performance of pest expert CNN model is similar with no misclassifications. This perfect invariance shows that pest subclasses have highly discriminative structural characteristics that are robust even under noise. The stability of the pest expert supports the concept that biologically limited classification decreases decision complexity while increasing robustness.

#### 5.4.4 Structural interpretation

The hierarchical evaluation indicates a consistent structural pattern. First, coarse biological routing in stage-1 is extremely stable across noise levels, maintaining around 98% accuracy even under severe perturbation. Second, intra-category expert sub networks achieve near-perfect discrimination inside their respective domains, with fungal and pest experts maintaining accuracy levels close to 100%. Third, perturbation sensitivity is significantly reduced compared to the flat CNN model.

Under high gaussian noise (*σ* = 0.05), the accuracy of flat CNN model dropped to 41%, whereas the hierarchical model retained stable performance (Stage-1 ≈ 98%, fungal expert ≈ 98%, pest expert = 100%). This stability shows that breaking down the classification into biologically meaningful stages simplifies decision boundaries and reduces cross-category interference. By separating inter-category routing from intra-category specialization, the hierarchical framework decreases the burden on a single model to learn complicated links between etiologically distinct diseases. Such architectural breakdown may help to improve robustness during distributional shift.

### 5.5 Overall hierarchical framework performance

#### 5.5.1 Overall classification performance

The whole hierarchical model, which combines stage-1 biological routing with stage-2 expert subnetworks, achieved overall test accuracy of 97.8% under clean conditions. The macro-averaged *F*1-score was 0.972, and the weighted *F*1-score was 0.978, demonstrating equal performance across all ten disease classes. Table 5 lists the classification performance metrics of overall hierarchical model. Minor confusion exists between early_blight and late_blight, which are visually similar fungal subclasses, but there is no systematic inter-category misrouting observed. The strong class-wise performance assures that hierarchical routing does not cause significant error propagation.

**Table 5:** Performance of hierarchical framework under clean and noisy conditions.

Metric	Clean	*σ* = 0.02	*σ* = 0.05
Accuracy	0.978	0.976	0.974
Macro precision	0.976	0.974	0.970
Macro recall	0.969	0.966	0.961
Macro *F*1-score	0.972	0.969	0.964
Weighted *F*1-score	0.978	0.976	0.974
Test samples	2,180	2,180	2,180

Under mild gaussian perturbation (*σ* = 0.02), overall accuracy decreased slightly to 97.6%, with a macro *F*1-score of 0.969. This reveals the low structural diversion from the clean condition, demonstrating that both routing model and expert sub networks are highly robust. Under higher perturbation (*σ* = 0.05), the hierarchical model maintained an accuracy of 97.4%, with a macro *F*1-score of 0.964 and weighted *F*1-score of 0.974. The degradation compared to clean conditions is only 0.4%, indicating outstanding stability of the hierarchical model under distributional shift. Table 5 presents the performance of the hierarchical model under clean and noisy perturbations.

Unlike the flat CNN model, which collapsed catastrophically under higher perturbations, the hierarchical model maintained consistent decision boundaries. The confusion matrices across noise levels remain structurally similar, with slight differences in intra-category confusion among visually similar fungal diseases. Most significantly, no widespread cross-category misclassification was identified. These findings show that breaking down classification into biologically meaningful stages efficiently lowers global interference across etiologically distinct diseases. By distinguishing between inter-category discrimination (Stage-1) from intra-category specialization (Stage-2), the model achieves both high discriminative performance and robustness.

The corresponding confusion matrices are provided in Supplementary Figure S6.

#### 5.5.2 Error propagation analysis

One possible constraint of hierarchical classification systems is the propagation of routing errors from the first-stage category classifier to the corresponding expert networks. To measure this effect, an error propagation analysis was performed on the test set. As a result, the proposed framework produces 58 final classification errors out of 2,180 test samples. Out of 58 errors, 42 errors (72.41%) were caused by incorrect category routing at stage 1, whereas 16 errors (27.59%) were from expert-network misclassification after correct routing. Table 6 lists the results of complete error propagation analysis.

**Table 6:** Error propagation analysis of the proposed hierarchical framework.

Error source	Number of errors	Percentage of final errors (%)
Stage-1 routing errors	42	72.41
Expert network errors	16	27.59
Total final errors	58	100.00

These findings suggest that routing model decisions are the principal cause of residual error in the hierarchical framework. However, the stage-1 routing model achieved a test accuracy of 97.98%, which reduced the amount of mis-routed samples. Furthermore, the expert networks retained strong classification performance within their respective biological categories, indicating that disease discrimination after correct routing is highly reliable. Therefore, future improvements in routing accuracy are expected to yield the largest gains in overall hierarchical classification performance.

#### 5.5.3 Interpretability analysis using grad-CAM

To check the biological feasibility of proposed hierarchical framework, gradient-weighted class activation mapping (Grad-CAM) was used to display the image areas that contribute most significantly to disease predictions. Grad-CAM visualizations were created for representative samples from both fungal and pest-related disease categories utilizing the corresponding category-specific expert networks.

**Figure 3:**
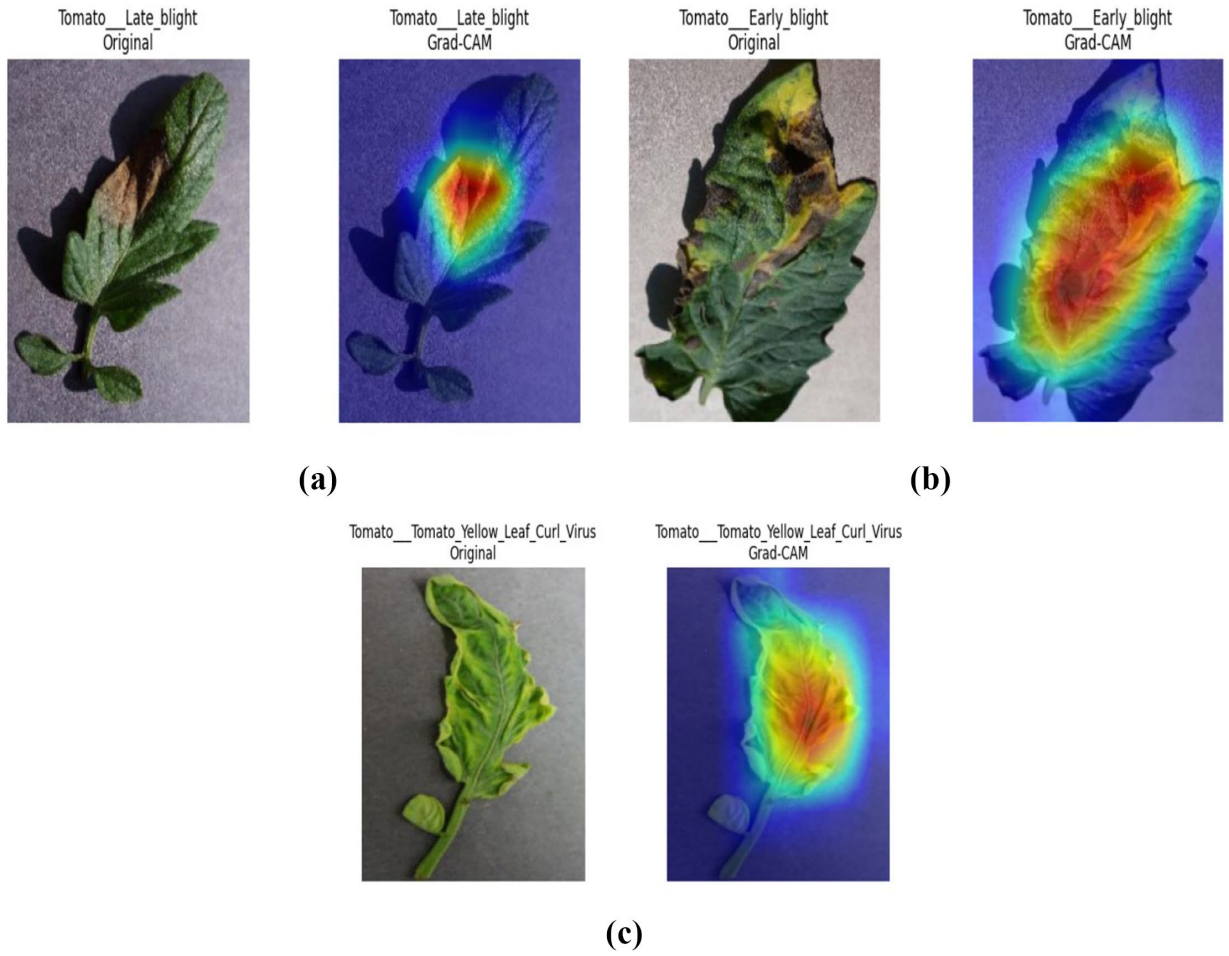
Grad-CAM visualizations for the representative disease categories processed by the category-specific expert networks. **(a)** Late blight, **(b)** early blight, and **(c)** tomato yellow leaf curl virus (TYLCV).

Figure 3 shows that the expert models consistently focus on diseased leaf regions rather than background areas. The activation maps of fungal diseases like early blight and late blight, highlights lesion clusters, necrotic regions and visibly infected tissue. Similarly, for pest-related diseases like tomato yellow leaf curl virus, the model concentrates on damaged and discolored leaf regions that correspond to underlying symptoms.

As per the observed activation patterns, the expert networks learn biologically meaningful visual representations that correspond to known disease characteristics. These results support the interpretability of proposed framework and gives evidence that the decision of prediction is primarily driven by disease-related features and not by irrelevant background information.

### 5.6 Comparative analysis of flat and hierarchical models

#### 5.6.1 Classification and robustness comparison

In addition to accuracy, balanced accuracy, Matthews Correlation Coefficient (MCC), and Cohen’s kappa are measured to give deeper evaluation. As shown in Table 7, all three models obtained high scores across these alternative evaluation metrics, indicating a high degree of agreement between predicted and true labels. The results also shows that the observed performance characteristics are not entirely dependent on overall accuracy.

**Table 7:** Performance comparison of flat and hierarchical models.

Model	Accuracy (%)	Balanced accuracy (%)	Accuracy *σ* = 0.02 (%)	Accuracy *σ* = 0.05 (%)	MCC	Kappa	95% confidence interval^*^	Performance drop (%)
CNN (flat)	99.3	99.37	92.3	41.0	0.9935	0.9935	99.14–99.76	−58.3
Vision transformer (flat)	96.3	96.90	96.2	94.8	0.9656	0.9655	96.36–97.77	−1.5
Hierarchical (ViT + CNN experts)	97.8	96.71	97.6	97.4	0.9688	0.9688	96.66–98.02	−0.4

^*^95% confidence intervals were calculated from the re-evaluated saved models and are reported to provide an estimate of statistical uncertainty.

Though Cohen’s kappa was provided as a extra agreement metric, its results should be evaluated with caution in imbalanced multi- class datasets, because they may be influenced by class prevalence. Thus, balanced accuracy and Matthews correlation coefficient (MCC) are believed to offer more thorough and trustworthy evaluation of model performance (Delgado and Tibau, 2019).

The comparison study reveals a clear trade-off between clean-test accuracy and robustness stability. The flat CNN model outperformed all the models in metrics like accuracy, balanced accuracy, MCC, and Cohen’s kappa values. However, when Gaussian perturbations increased, its performance dropped significantly, indicating a strong sensitivity to noise. But image augmentation was done for flat CNN model which is typically used during training phase of the model. So, this limitation should be taken into consideration during interpretation of results. In contrast, the flat ViT model maintained a more stable performance under perturbation through global self-attention-based feature modeling.

**Figure 4:**
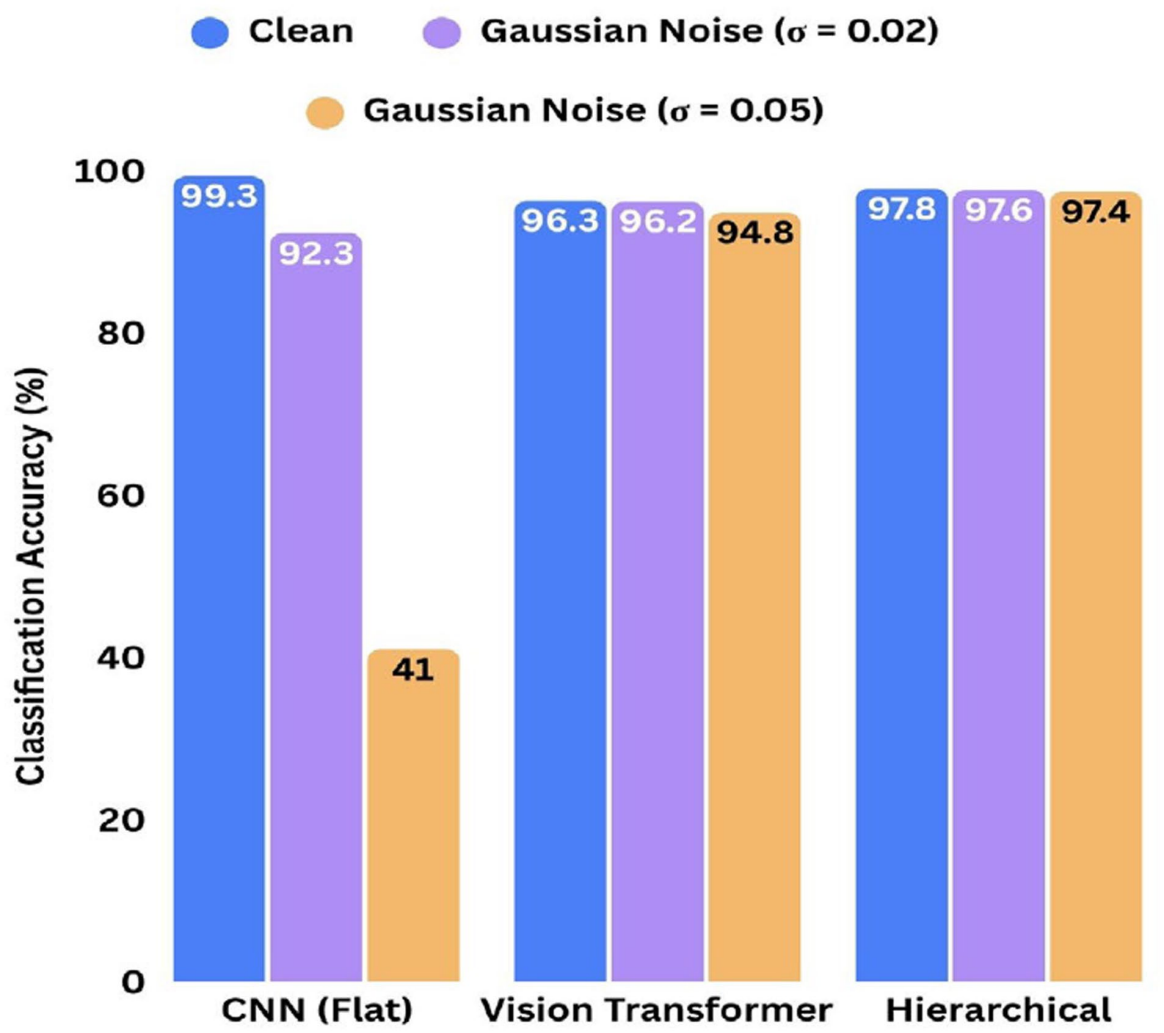
Robustness comparison of flat CNN, flat ViT and hierarchical framework under increasing Gaussian noise levels.

The proposed hierarchical model performed competitively clean-test performance while exhibiting the highest robustness across different Gaussian noise levels. By combining biologically guided routing with category-specific expert specialization, the framework reduced performance degradation to only 0.4% under strong perturbation (*σ* = 0.05). Figure 4 depicts the robustness degradation of the flat CNN model, flat ViT model and hierarchical model under different Gaussian perturbations. These findings imply that robustness can be improved not only through architectural design but also through biologically informed structural decomposition of the classification task.

To further strengthen the statistical evaluation, 95% confidence intervals were calculated for the classification accuracy of all models. As shown in Table 7, the resulting intervals are narrow, demonstrating stable performance estimates and confirms the reliability of the obtained results.

#### 5.6.2 Computational efficiency trade-off analysis

In addition to classification accuracy and robustness, computational efficiency was calculated by taking the average inference time per image, the number of trainable parameters, model memory footprint and computational complexity for all architectures. This study reveals the practical feasibility of using proposed framework in real-world agricultural diagnostic systems, where prediction accuracy and computational cost are crucial factors.

As shown in Table 8, the flat CNN model has 5.29 million parameters and requires 410.20 MMac, whereas the flat ViT model contains 5.72 million parameters and requires 912.89 MMac. The proposed hierarchical model, which consists of stage-1 ViT routing model and two category-specific EfficientNet-B0 expert networks, contains a total of 16.30 million parameters and occupies 62.47 MB of memory. It also lists average inference time used by the flat CNN model, flat ViT model and hierarchical model. The flat CNN model took about 65.4 milliseconds (ms) per image, whereas the flat ViT model took about 63.0 ms per image. The proposed hierarchical model required 75.2 ms per image. This shows that the inclusion of the stage 1 ViT routing model followed by a category-specific expert CNN model results in a slight additional computational cost, which is fair trade-off between model performance and computation.

**Table 8:** Comparison of computational efficiency of flat and hierarchical models.

Model	Parameters (*M*)	Model size (MB)	MACs (MMac)	Accuracy (%)	Inference time (ms/image)
CNN (EfficientNet-B0)	5.29	20.33	410.20	99.3	65.4
Vision transformer (DeiT-Tiny)	5.72	21.81	912.89	96.3	63.0
Hierarchical (DeiT router + expert CNNs)	16.30	62.47	1733.29	97.8	75.2

However, it is essential to remember that during inference only expert network related with corresponding predicted biological category is only activated. As a result, the hierarchical architecture acts as a conditional routing system rather than executing all expert networks at the same time. This implies that the computational cost remains low while improving robustness and reducing inter-class inference. This small computational overhead is accompanied by significantly improved robustness under Gaussian perturbation, indicating that the proposed biologically structured decomposition provides a practical trade-off between computational cost and classification reliability.

### 5.7 Qualitative end-to-end inference

**Figure 5:**
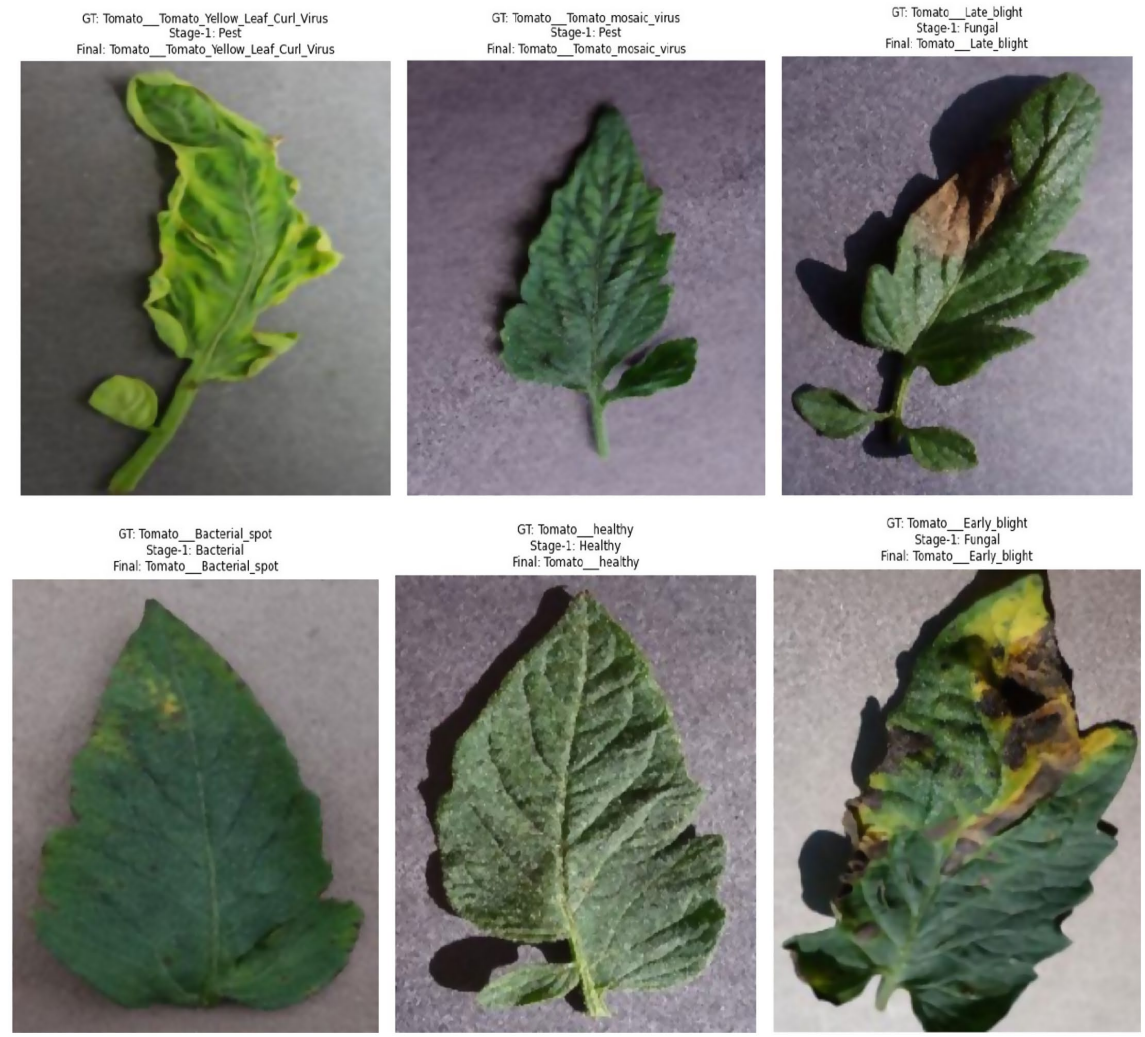
Representative examples of end-to-end hierarchical inference showing ground truth (GT), stage-1 biological category prediction and final class output for selected test samples.

To validate the hierarchical framework’s end-to-end behavior, representative test images were run through the whole two-stage pipeline. Figure 5 shows the examples illustrating ground truth (GT), stage-1 biological routing output and final fine-grained prediction.

In all the tested samples, the routing stage properly identified the biological category (bacterial, fungal, pest-associated, or healthy) and the corresponding expert network also generated the correct final disease label. These examples confirm that the hierarchical model conducts coherent coarse-to-fine reasoning on representative samples, with no obvious routing failure. The structured inference approach improves interpretability by clearly distinguishing biological category identification from subclass discrimination.

The results of this study are consistent with recent developments in deep learning based plant disease classification in which high classification accuracy has been shown by CNN, vision transformer and hybrid architectures on benchmark datasets (Karthikeyan et al., 2025; Arshad et al., 2026). But still, the majority of current research focus on enhancing network architectures or feature extraction within a flat classification network. So, the suggested approach differs from this regular trend by presenting a biologically structured hierarchical classification strategy that performs fine-grained classification by primarily categorizing diseases based on their biological etiology. By reducing inter-category confusion and retaining great robustness during distribution shift, this approach creates a trustworthy plant diagnosis system which is appropriate for real-world agricultural applications.

## 6 Conclusion

This paper developed a biologically structured hierarchical deep learning architecture by combining transformer-based biological routing with CNN-based expert specialization for tomato leaf disease classification. Unlike traditional flat multi-class classification, the proposed approach divides classification into coarse biological categorization followed by fine-grained intra-category discrimination.

Experimental results on PlantVillage tomato dataset revealed that a flat CNN model achieved the highest clean accuracy (99.3%), but it showed significant degradation under Gaussian perturbation, with performance dropping to 41% at *σ* = 0.05. On the other hand, the flat ViT model maintained steady performance in the presence of Gaussian noise but acts as a flat classification structure. The proposed hierarchical model obtained 97.8% clean accuracy and maintained 97.4% accuracy under high perturbation, with only 0.4% of minimal performance degradation.

As per the stage-wise analysis, biological routing remained highly robust under Gaussian noise perturbations, whereas expert subnetworks achieved near-perfect intra-category discrimination. Without compromising clean accuracy, the hierarchical decomposition efficiently reduced inter-class interference and decision boundaries resulting in increased robustness.

These results suggest that reliability under distributional shift can be improved by incorporating domain-informed biological structure into model design. Compared to existing CNN and vision transformer approaches which mostly improves feature extraction within single stage classifier, the proposed biologically structured hierarchical framework reduces inter-category confusion by disease etiology routing and also remains highly robust to perturbations. So, in real-world agricultural environment where image variability is unavoidable, the proposed framework offers a reliable approach for tomato disease diagnosis.

## 7 Future work

The proposed framework is resistant to significant Gaussian perturbations, however, further research has to be carried out in certain areas. First, it is crucial to evaluate the model under real-world agricultural field conditions. The current experiments were carried out on the controlled PlantVillage dataset, but future research should compare performance to field acquired images with varied background, illumination variations and occlusions.

Second, though image augmentation is primarily used to increase robustness against distribution shifts, flat CNN model was trained without it in this study. So, the robustness comparison between hierarchical framework and flat CNN model could not be completely equal assessment. Future research should use consistent augmentation technique for all baseline models and also robustness evaluation should include illumination variations, blur, compression artifacts, occlusions, contrast changes, sensor variability and domain-shift scenarios between controlled and field environments. Such investigations would provide a more comprehensive assessment of deployment stability under real-world agricultural conditions.

Third, to reduce the potential errors from stage-1 routing, adaptive routing mechanisms should be studied. Reliability can be further enhanced by probabilistic routing or confidence-based expert selection. Furthermore, although the hierarchy employed in this study was based on expert-defined biological categories for tomato diseases, the proposed framework is not crop-specific. The same hierarchical decomposition method can be used to other agricultural datasets through appropriate disease grouping strategies. Future study may also investigate automatically learned hierarchical structures and mechanisms for handling diseases with overlapping or unclear biological characteristics. Finally, using the biologically structured hierarchical technique to multi-crop disease datasets could demonstrate its generalizability and scalability for broader agricultural diagnostic applications.

## Data Availability

Publicly available datasets were analyzed in this study. This data can be found here: https://www.kaggle.com/datasets/charuchaudhry/plantvillage-tomato-leaf-dataset.
