# Supplementary material for: A biologically structured hierarchical vision transformer-CNN framework for robust tomato leaf disease classification
Source: Front Artif Intell. 2026 Aug 5;9:1880070. doi: 10.3389/frai.2026.1880070 (PMC13485869; doi:10.3389/frai.2026.1880070)
Supplement: Supplementary file 1 [file Image_1.PDF]

Supplementary Material

Supplementary Figure S1

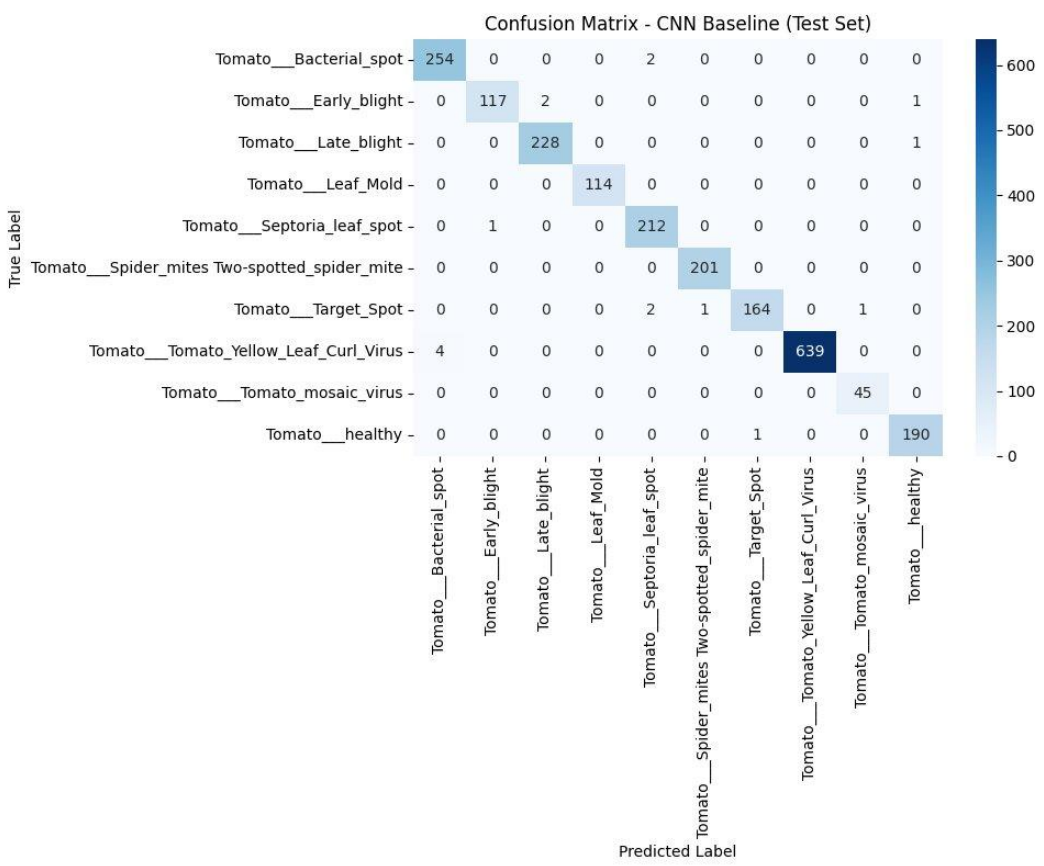

(a)

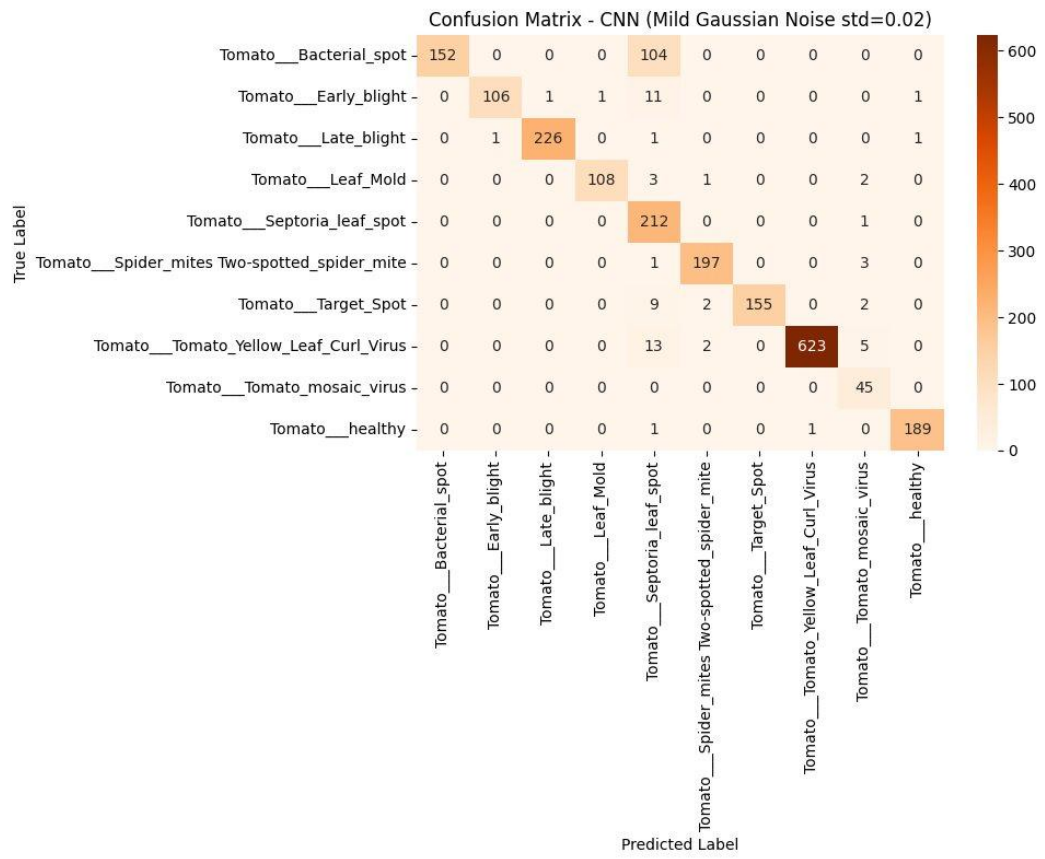

(b)

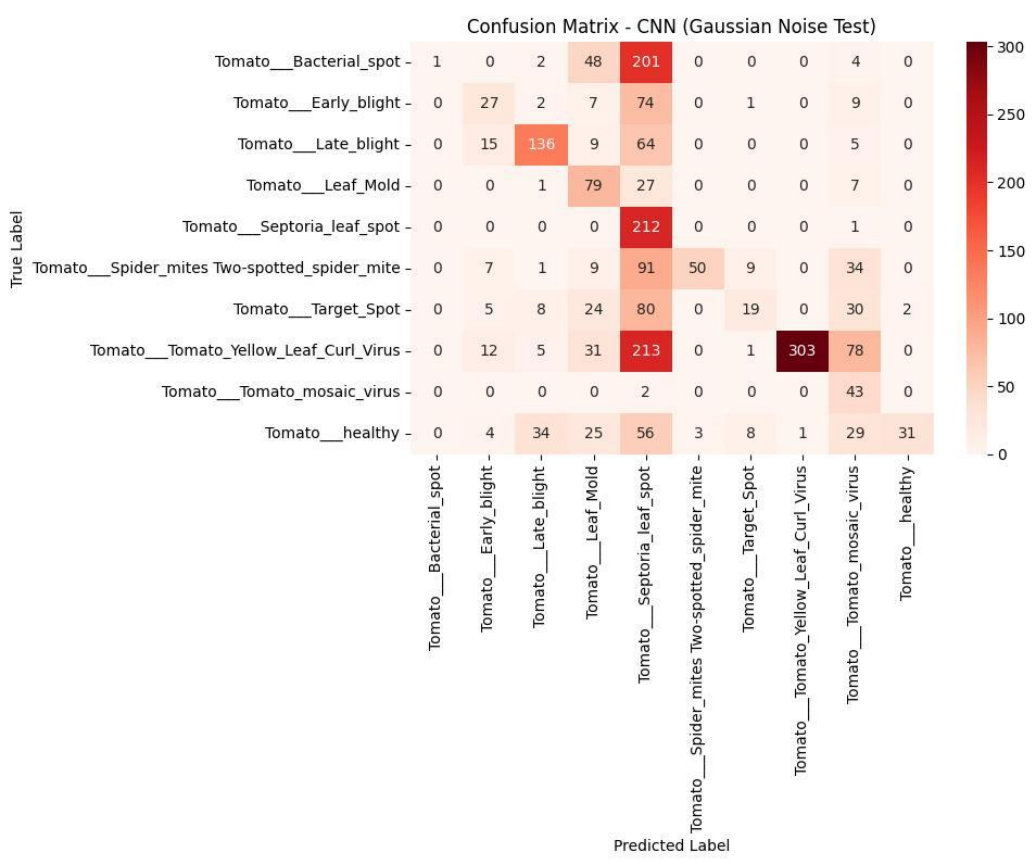

(c)

Supplementary Figure S1. Confusion matrices of the flat CNN model under (a) the clean test set, (b) Gaussian noise with σ = 0.02 and (c) Gaussian noise with σ = 0.05

Supplementary Figure S2

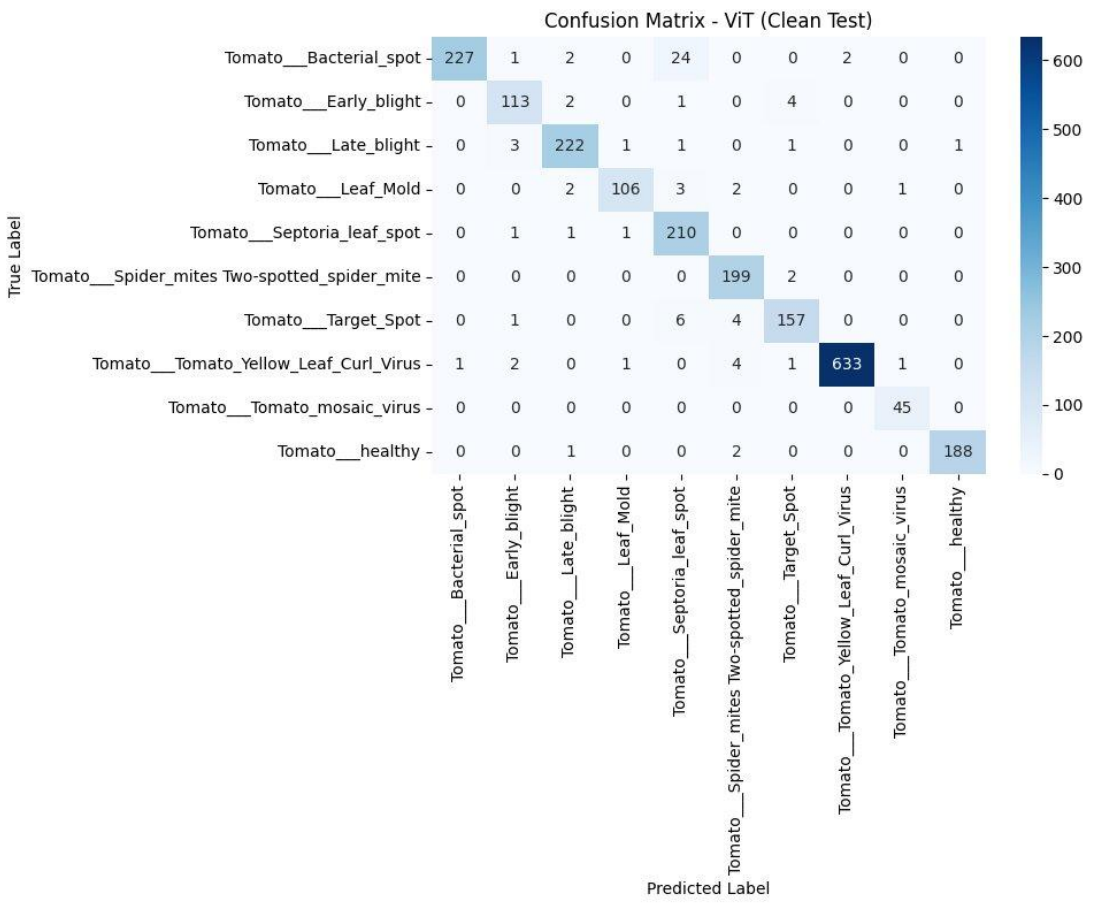

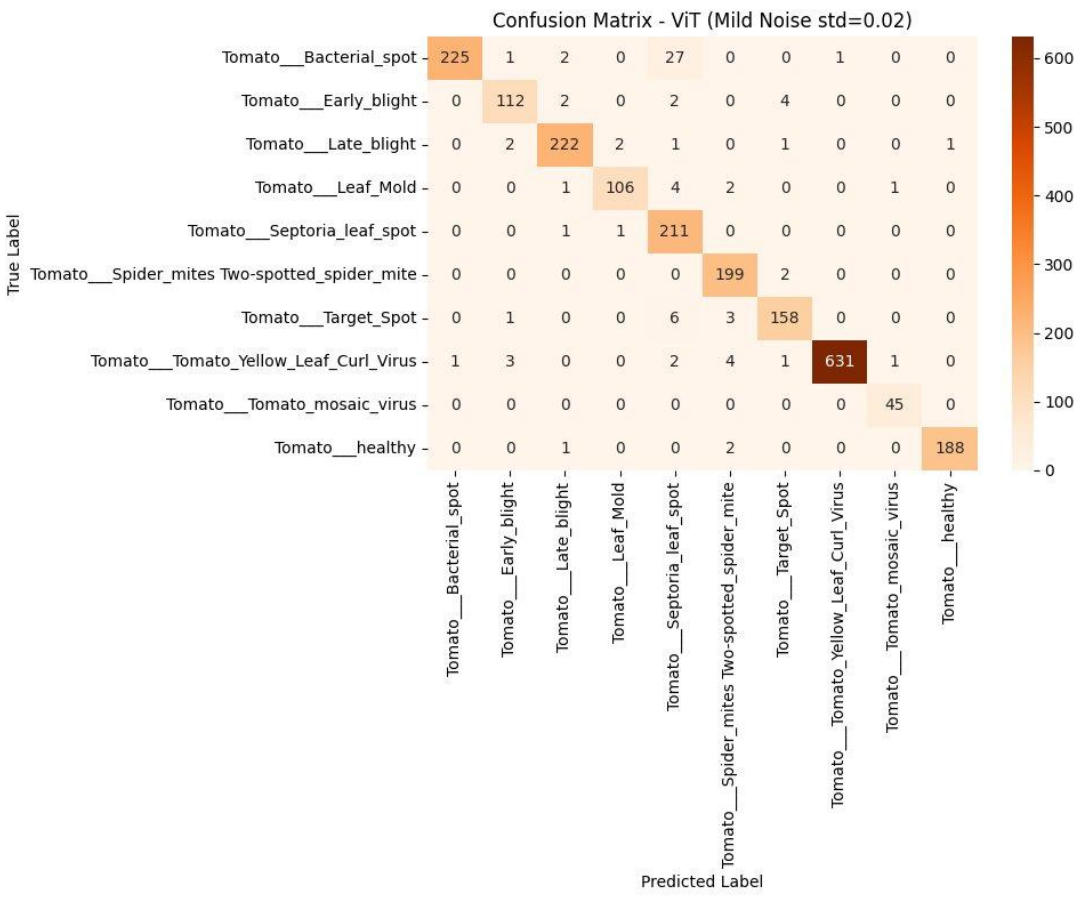

(a) *(b)*

Supplementary Figure S2. Confusion matrices of the flat ViT model under (a) clean test condition and (b) Gaussian noise with σ = 0.02. (The confusion pattern for σ = 0.05 was similar and is omitted for brevity)

Supplementary Figure S3

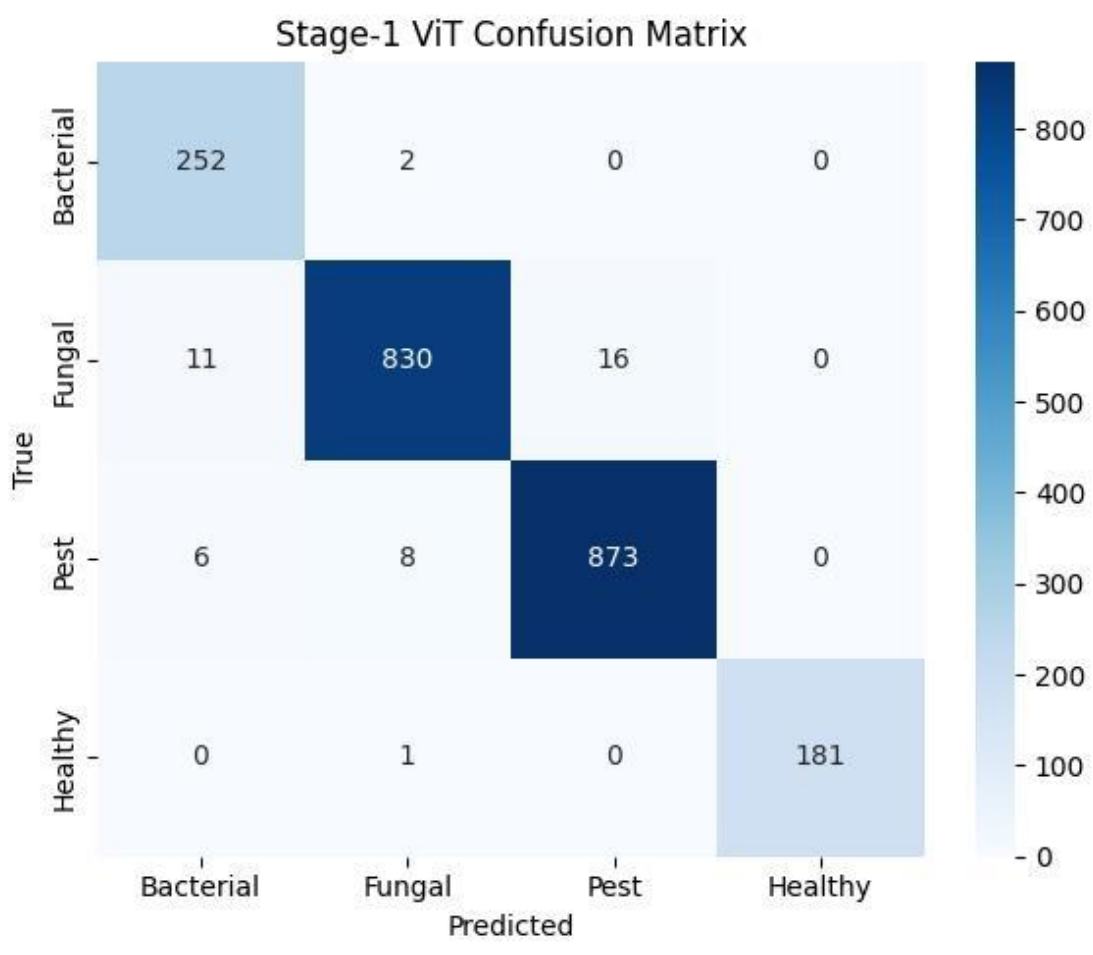

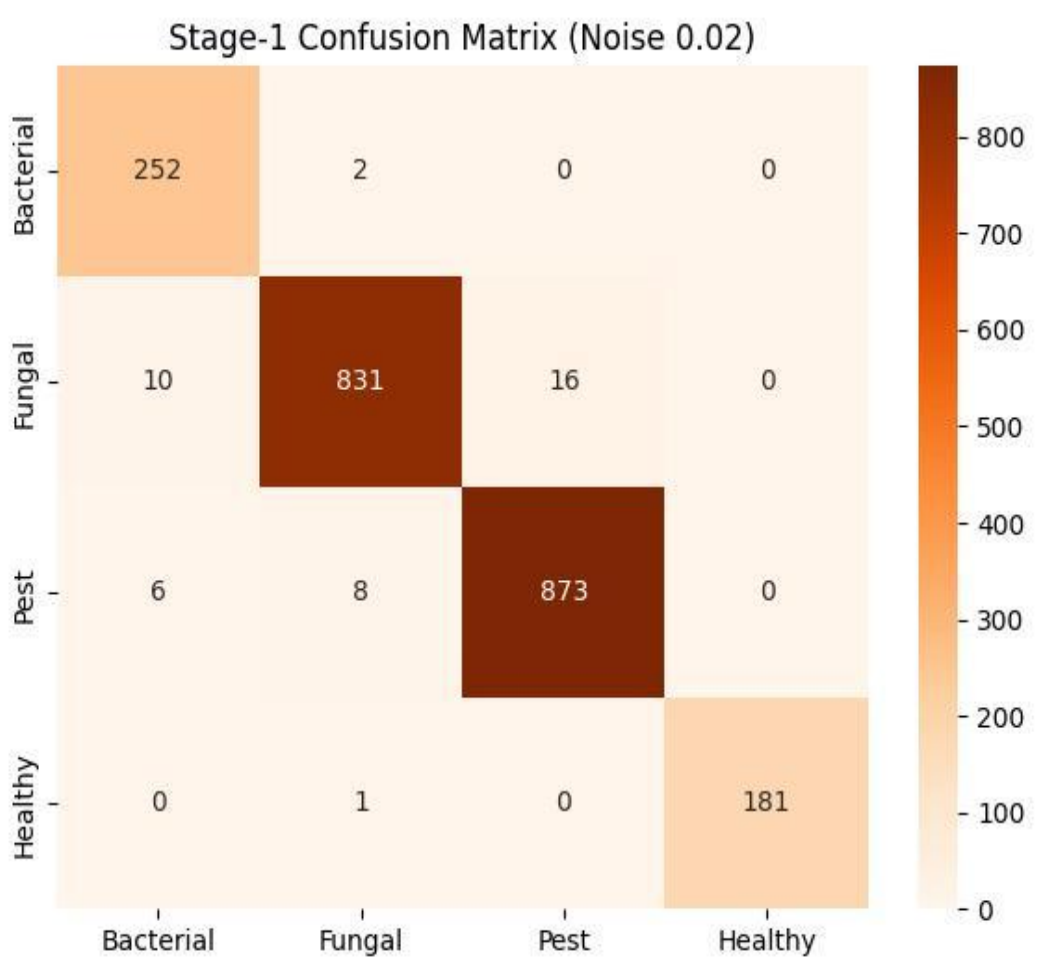

(a) *(b)*

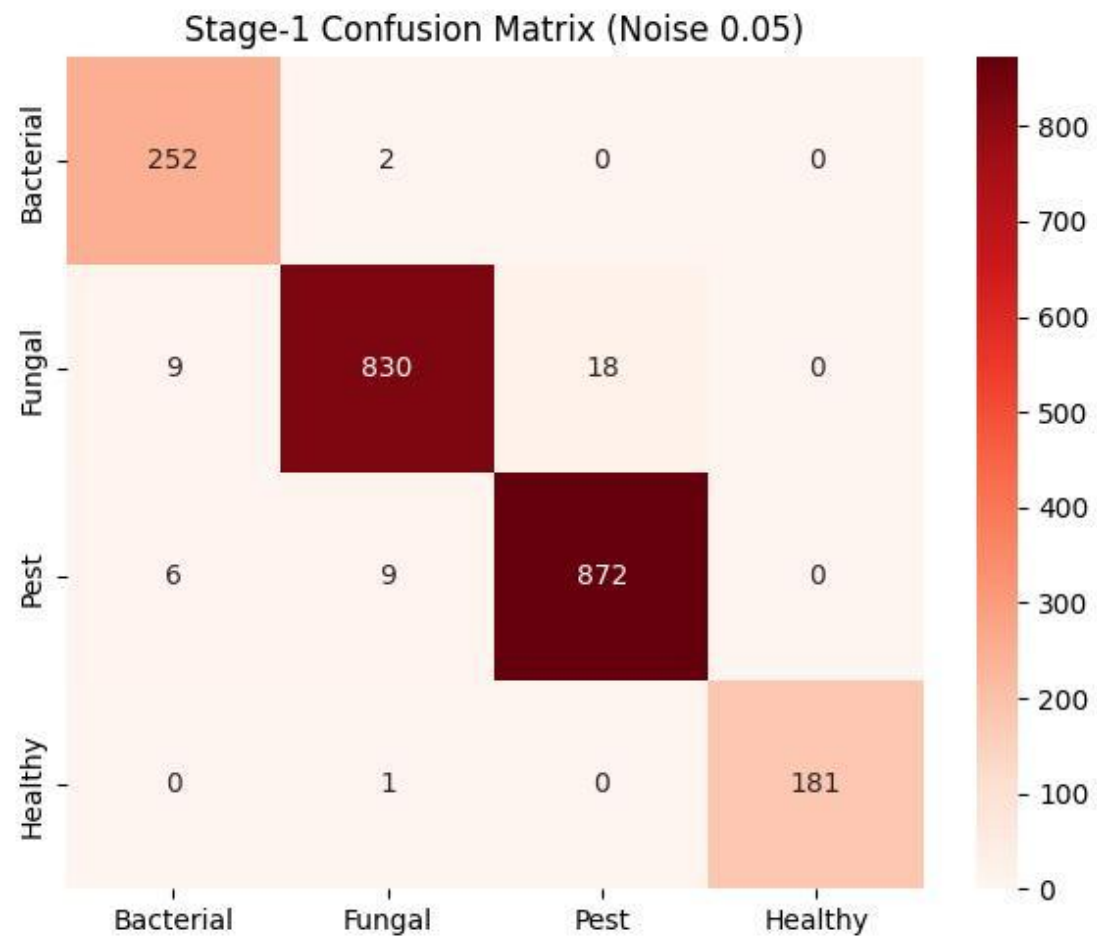

(c)

Supplementary Figure S3. Confusion matrices of the stage-1 ViT routing model under (a) the clean test set, (b) Gaussian noise with σ = 0.02, and (c) Gaussian noise with σ = 0.05.

Supplementary figure S4

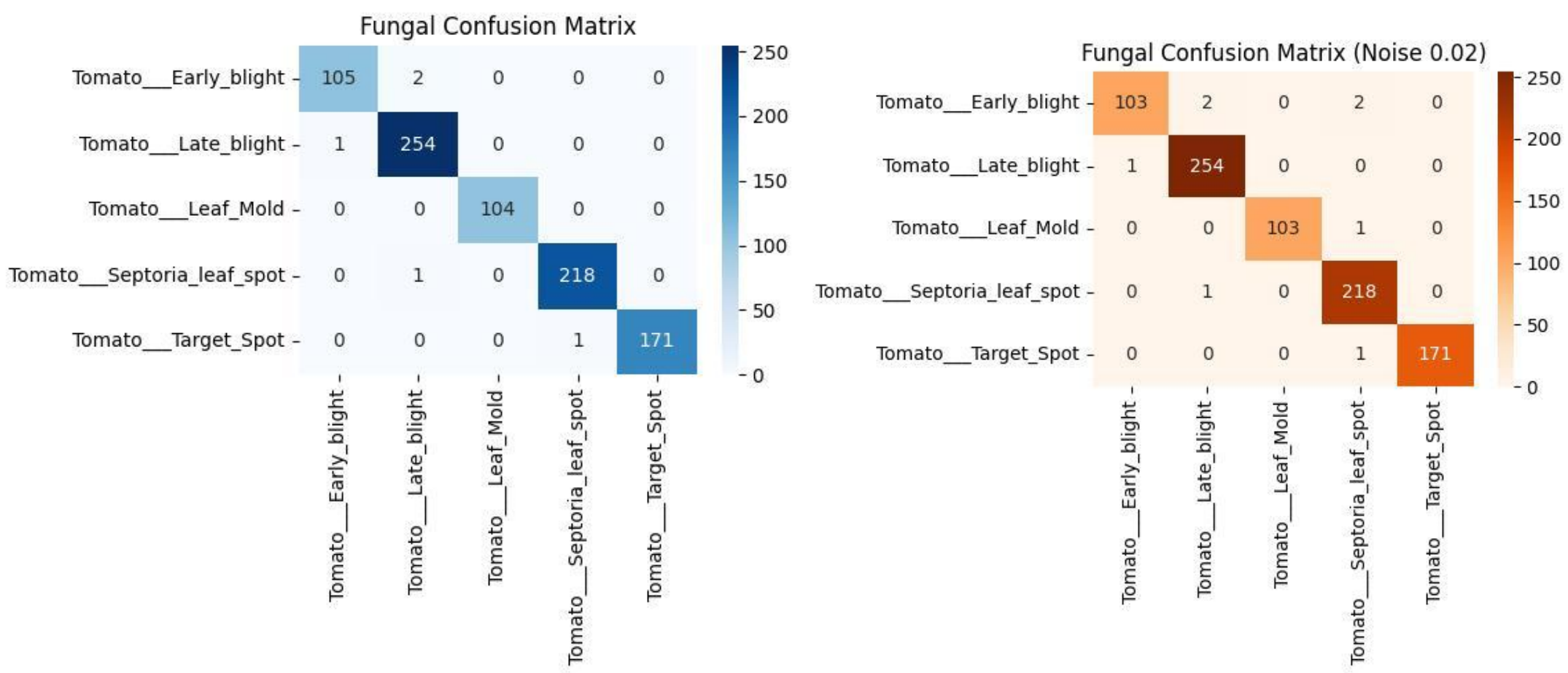

(a) *(b)*

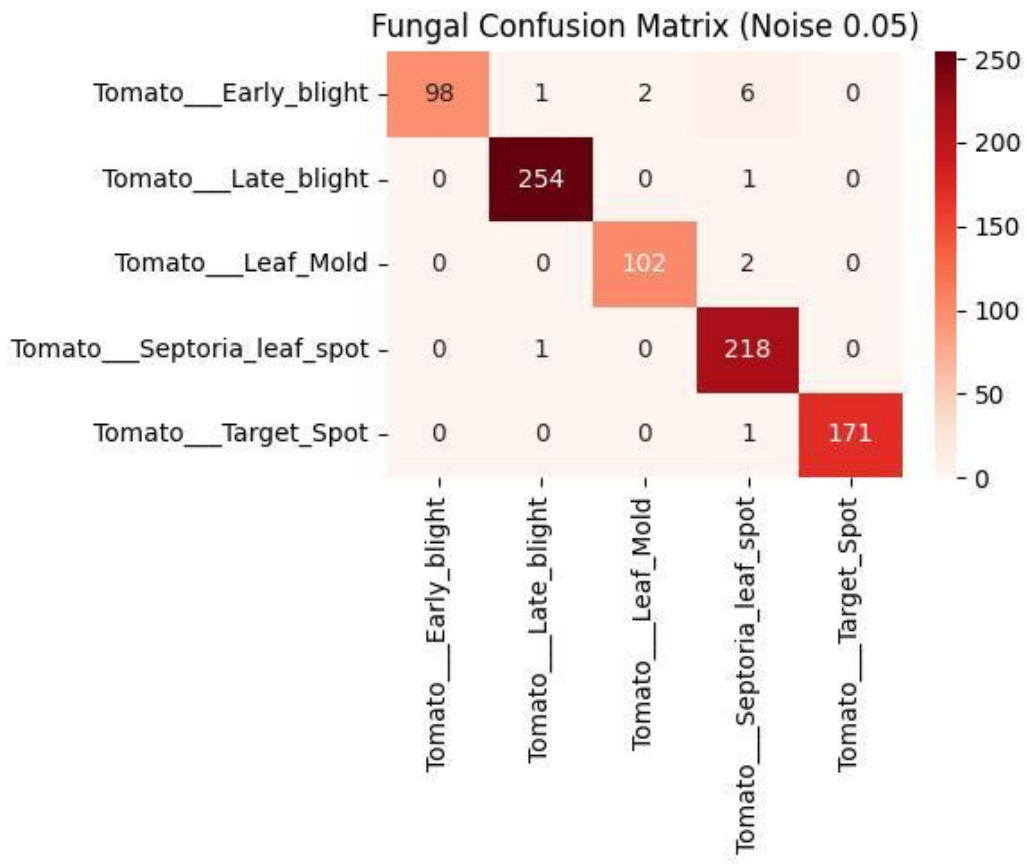

(c)

Supplementary Figure S4. (a) Confusion matrix of fungal expert CNN model (b) Confusion matrix of fungal CNN model under 0.02 gaussian noise (c) Confusion matrix of fungal CNN model under 0.05 gaussian noise

Supplementary Figure S5

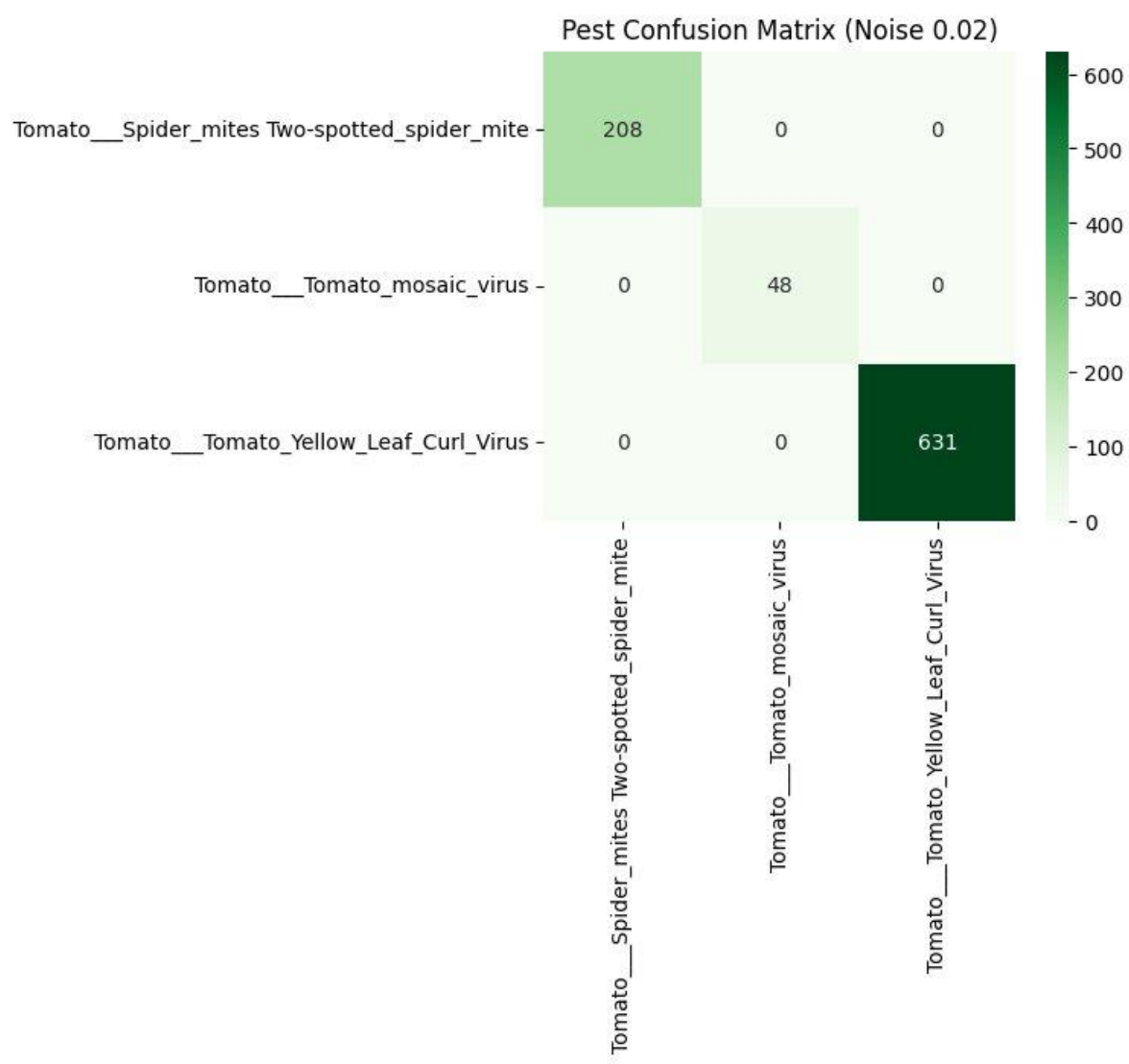

Supplementary Figure S5. Confusion matrices of the pest expert CNN model under the clean test condition (Performance remained identical across perturbation levels, so only the clean confusion matrix is shown for brevity)

Supplementary Figure S6

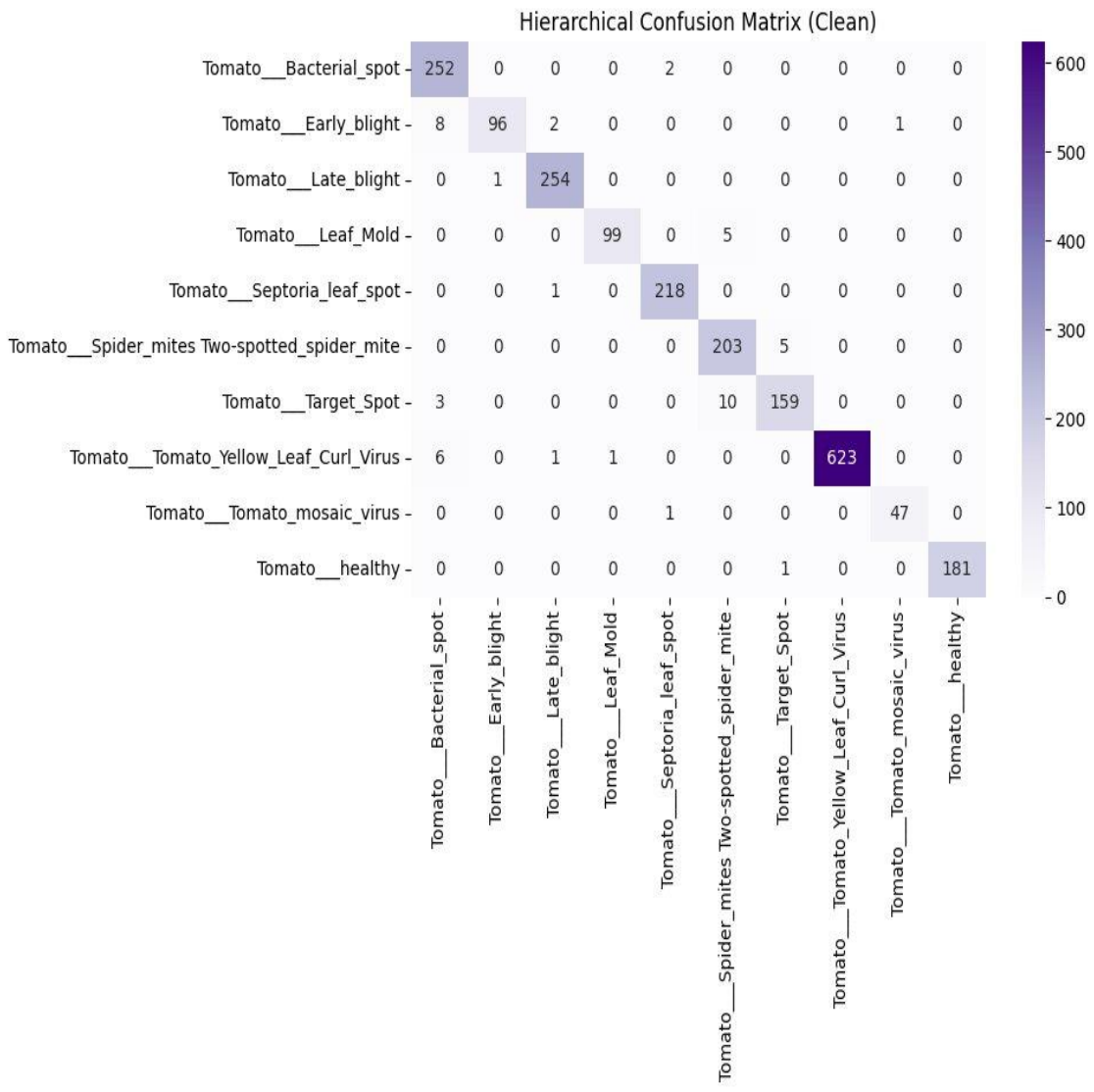

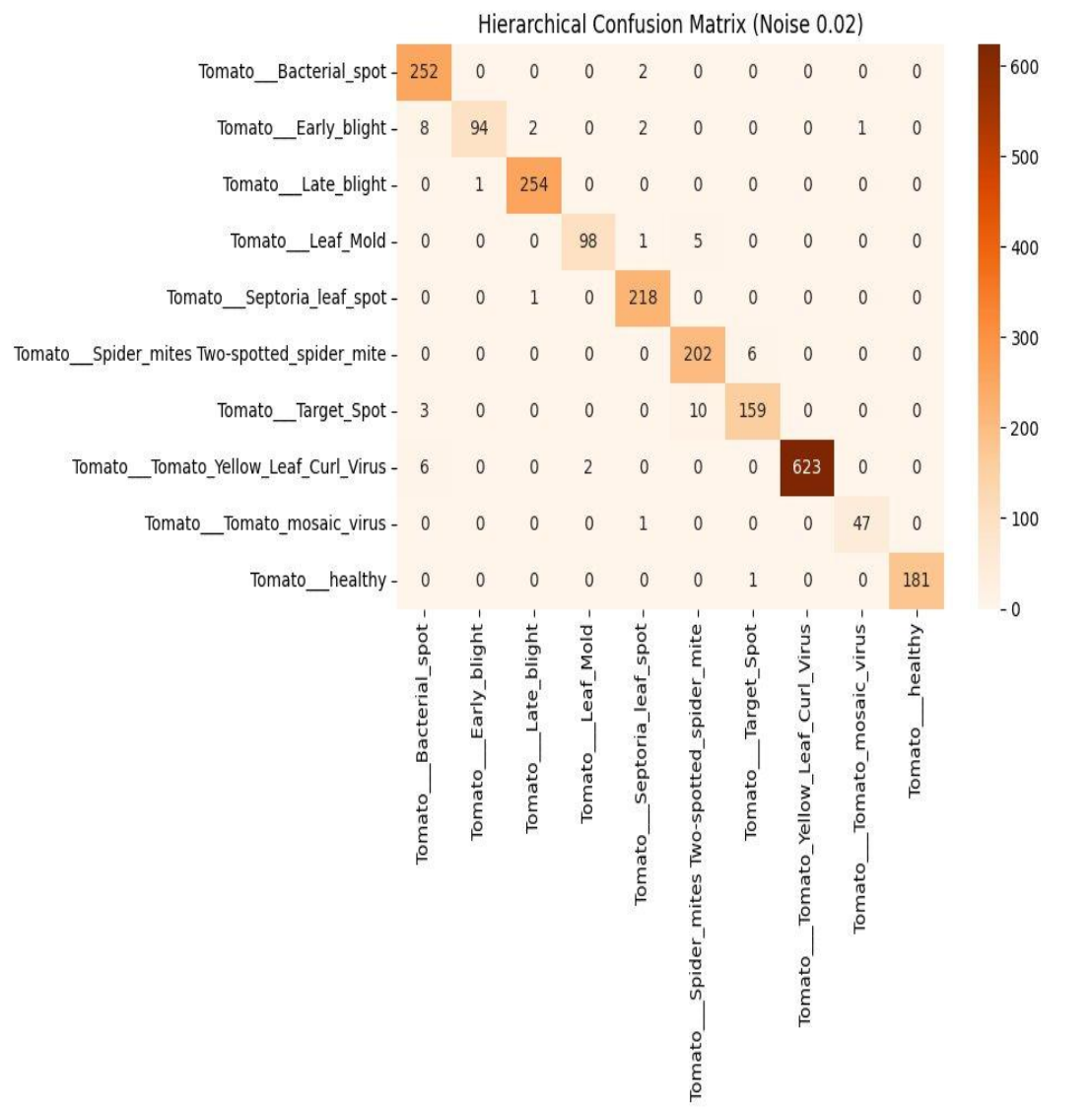

(a) *(b)*

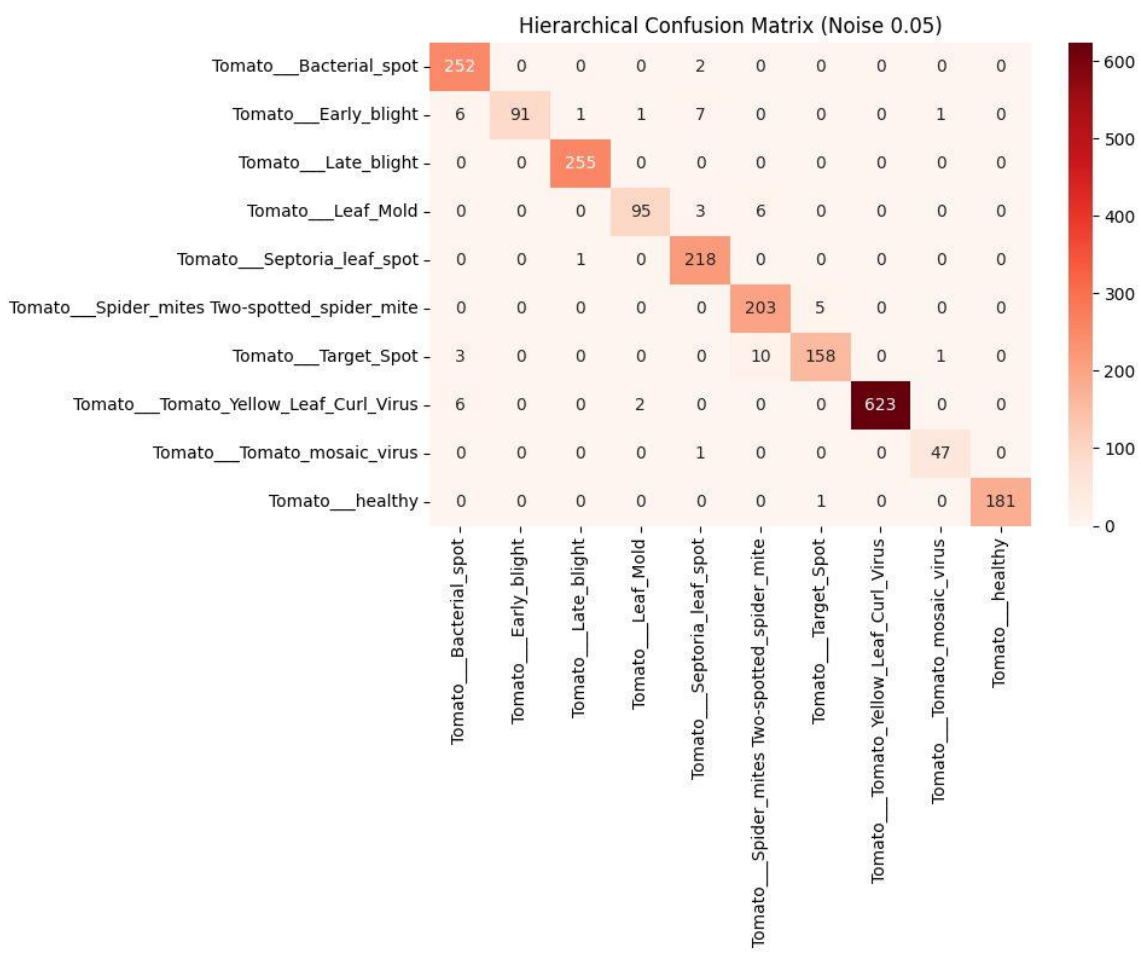

(c)

Supplementary Figure S6. Confusion matrices of the proposed hierarchical model under (a) the clean test set, (b) Gaussian noise with σ = 0.02, and (c) Gaussian noise with σ = 0.05.
